# Transcriptome Alterations of an *in vitro*-Selected, Moderately Resistant, Two-Row Malting Barley in Response to 3ADON, 15ADON, and NIV Chemotypes of *Fusarium graminearum*

**DOI:** 10.3389/fpls.2021.701969

**Published:** 2021-08-11

**Authors:** James R. Tucker, William G. Legge, Sujit Maiti, Colin W. Hiebert, Senay Simsek, Zhen Yao, Wayne Xu, Ana Badea, W. G. Dilantha Fernando

**Affiliations:** ^1^Department of Plant Science, University of Manitoba, Winnipeg, MB, Canada; ^2^Brandon Research and Development Centre, Agriculture and Agri-Food Canada, Brandon, MB, Canada; ^3^Morden Research and Development Centre, Agriculture and Agri-Food Canada, Morden, MB, Canada; ^4^Department of Plant Science, North Dakota State University, Fargo, ND, United States

**Keywords:** malt, Fusarium head blight, *Fusarium graminearum*, RNA-Seq, *in vitro* selection, deoxynivalenol

## Abstract

Fusarium head blight caused by *Fusarium graminearum* is a devastating disease of malting barley. Mycotoxins associated with contaminated grain can be transferred from malt to beer and pose a health risk to consumers. In western Canada, *F. graminearum* has undergone an adaptive shift from 15ADON constituency to dominance by virulent 3ADON-producers; likewise, NIV-producers have established in regions of southern United States. Lack of adapted resistance sources with adequate malting quality has promoted the use of alternative breeding methodologies, such as *in vitro* selection. We studied the low-deoxynivalenol characteristic of *in vitro* selected, two-row malting barley variety “Norman” by RNAseq in contrast to its parental line “CDC Kendall,” when infected by 15ADON-, 3ADON-, and NIV-producing isolates of *F. graminearum*. The current study documents higher mycotoxin accumulation by 3ADON isolates, thereby representing increased threat to barley production. At 72–96-h post infection, significant alterations in transcription patterns were observed in both varieties with pronounced upregulation of the phenylpropanoid pathway and detoxification gene categories (UGT, GST, CyP450, and ABC), particularly in 3ADON treatment. Defense response was multitiered, where differential expression in “Norman” associated with antimicrobial peptides (thionin 2.1, defensing, non-specific lipid-transfer protein) and stress-related proteins, such as late embryogenesis abundant proteins, heat-shock, desiccation related, and a peroxidase (*HvPrx5*). Several gene targets identified in “Norman” would be useful for application of breeding varieties with reduced deoxynivalenol content.

## Introduction

Fusarium head blight (FHB), caused by *Fusarium graminearum* Schwabe [teleomorph: *Gibberella zeae* (Schwein.) Petch], is an economically important disease of barley (*Hordeum vulgare* L.) that has resulted in massive commercial losses (Wilson et al., 2018). *F. graminearum* is capable of producing multiple, type B trichothecene mycotoxins [Deoxynivalenol (DON), 3-acetyldeoxynivalenol (3ADON), 15-acetyldeoxynivalenol (15ADON), nivalenol (NIV), and 4-acetylnivalenol (4-ANIV)], which can be commonly detected in contaminated grain (Miller et al., 1991). Type B trichothecenes are harmful to eukaryotic organisms through interference of ribosomal function and protein translation (Desjardins, 2006). Mycotoxin content in grain is highly scrutinized by the malting and brewing industries due to their potential human health concerns. Arising from their water-soluble nature, mycotoxins, such as DON, can be transferred from malt to beer (Habler et al., 2017), which may be of elevated concern for the increasingly popular all-malt, craft brewing industry (Peters et al., 2017). Extremely strict, industrial limits on mycotoxins (<0.5 mg kg^−1^) commonly result in loss of premiums, which would be received below prime sale value in the lucrative malting barley market.

Immune reaction has not been reported within barley, and resistance sources are extremely limited (Bai and Shaner, 2004). Furthermore, resistance breeding of malting barley through back-cross methodologies has been difficult due to strict requirements of numerous quality parameters. *In vitro* selection (*IVS*) of somaclonal variations, which employs tissue culture and growth media, containing selective agents, offers an alternative option, for example, to rapidly improve disease resistance in crops without linkage drag (Rao and Sandhya, 2016). This method is an acceptable biotechnological tool based on changes resulting from internal variations/mutations. Examples of *IVS*, using fusaric acid or mycotoxins as selective agents in barley (Chawla and Wenzel, 1987) and wheat (Eudes et al., 2008) have been reported. “Norman” is an *IVS* two-row, malting variety developed *via* anther culture, using media, containing 1.71-mg kg^−1^ DON, which displays reduced DON accumulation, without change in agronomics and malting quality. Based on DON content, it was evaluated with a moderately resistant reaction to FHB in the Western Cooperative Two-Row Barley Registration Test in 2005 and 2006, as part of the western Canadian registration recommending process under the auspices of the Prairie Registration Recommending Committee for Oat and Barley (Legge et al., 2011).

Several studies on wheat have indicated that 3ADON-producers are more aggressive and generally produce more DON than 15ADON-producers (Ward et al., 2008; Puri and Zhong, 2010; von der Ohe et al., 2010). Field-based studies of barley correlate 3ADON with higher DON content, implicating chemotype-associated virulence (Clear et al., 2013; Tucker et al., 2019). In a pan-genome study of 60 North American *F. graminearum* isolates, Kelly and Ward (2018) confirmed signatures of selection in the *TRI* (trichothecene) gene cluster region, signifying importance of this region in genomic divergence. Moreover, analysis of gene expression through RNA-sequencing (RNA-Seq) further demonstrated differential gene expression between 3ADON and 15ADON-producers, notably in metabolic function and trichothecene production (Walkowiak et al., 2015; Puri et al., 2016). Amarasinghe and Fernando (2016) observed elevated *TRI* gene expression in 3ADON-producers in contrast to 15ADON or NIV strains during infection of wheat. Al-Taweel et al. (2014) documented differential transcription patterns in wheat challenged by 15ADON or 3ADON strains, where 3ADON-producers suppressed host resistances. Comparative studies can help elucidate underlying mechanisms of aggressiveness and contribute toward understanding of plant defense response.

*Fusarium graminearum* displays a complex lifestyle (hemi-biotrophism), where it initially lives in a biotrophic stage, asymptomatically within the apoplast for a few days, followed by a prolific stage under a switch to necrotrophy accompanied by elevated mycotoxins used to kill host tissues for consumption (Trail, 2009). DON is not essential for infection, as demonstrated by loss-of-function trichothecene non-producing mutants (*TRI5*-), which are still capable of infecting barley (Jansen et al., 2005; Boddu et al., 2007). Yet, DON is a virulence factor where DON-expressing wild-type strains cause higher disease severity than *TRI5*- mutants (Boddu et al., 2007). A critical phase of host-response occurs at ~72 h post-infection (hpi), associated with an upsurge in the rate of DON production (Boddu et al., 2006, 2007). Study of *F. graminearum* displays a reciprocal peak of gene activity at 72 hpi, where genes of cell wall-degrading enzymes and trichothecene biosynthesis are highly expressed (Güldener et al., 2006). This unique phase of host–pathogen interaction is distinguished by pathogen transition to necrotrophy.

While quantitative disease resistance may contribute to deployment of durable varieties, this form of resistance remains poorly understood (Poland et al., 2009). Herein, we study complex disease resistance through contrast of moderately resistant (MR) variety “Norman” to its parent line “CDC Kendall” (intermediately resistant, MR-MS), which carries both resistant and susceptible features. Compared with major-gene resistance, less is known about mechanisms of quantitatively inherited disease resistance. The transcriptome differentiation landscape would provide insight into the FHB resistance responses. Although in recent years, RNA-Seq has become a powerful tool for identification of genes associated with differential transcription patterns (Wang et al., 2009), this technology has not yet been applied to analyze the resistance response of two-row barley to *F. graminearum*. The objective of this study was to detect and quantify transcriptional differences between moderately resistant, *IVS* variety “Norman,” and its intermediately resistant parent variety “CDC Kendall” when challenged by differentially virulent *F. graminearum* chemotypes. Together, the study provides insight into expression of genomic features of DON resistance in “Norman” and its interaction with pathogens characterized by differential production of secondary metabolites, such as trichothecenes.

## Materials and Methods

### Long-Term DON Content Evaluation of “Norman” vs. “CDC Kendall”

The varieties evaluated in this study were: “Norman” and “CDC Kendall.” “Norman” is an *IVS* variety developed under joint release between Agriculture and Agri-Food Canada, Brandon Research and Development Centre (AAFC-Brandon) and Crop Development Centre (CDC), University of Saskatchewan (Legge et al., 2011). “Norman” was derived from “CDC Kendall,” a malting variety developed by CDC^1^ The data used for this evaluation were generated during a 20-year period, 2001–2020. Briefly, the varieties were grown in 0.9-m rows in an irrigated FHB nursery at AAFC-Brandon, MB, as outlined in Legge et al. (2011). DON content was determined on 20-g subsample of matured grain was ground, using a Perten 3600 laboratory mill. From this, a 1-g sample was used in assay of DON content determination, following the enzyme-linked immunosorbent assay (ELISA) method (Sinha and Savard, 1996). Least squares means were calculated for DON content of each variety within site year. To normalize data, a logarithmic transformation (LOG_10_) was applied to the DON data prior to analysis. A matched pairs procedure was applied to test for mean difference of DON content between varieties (SAS JMP® 13.2.1, SAS Institute Inc. Cary, NC, USA, SAS Institute Inc, 1989-2019).

### Genotyping

Approximately 100 mg of fresh leaf tissue was sampled from seedlings of each variety, flash frozen in liquid N_2_, and then freeze-dried. DNA was extracted, using Qiagen, DNeasy 96 Plant Kit (Qiagen, Canada), as per the handbook of the manufacturer and then normalized to 50 ul ml^−1^. Samples were genotyped with an Illumina iScan (Illumina, San Diego, CA, USA), using an Infinium HTS iSelect−50 K SNP custom microarray (Bayer et al., 2017). Chromosome graphics were produced, using R package “chromoMap” (Anand and Rodriguez Lopez, 2020).

### Fungal Cultures

Three chemotypes were chosen to be used in this study: 15ADON, 3ADON, and NIV, with four single-spore isolates per chemotype group. The *F. graminearum* isolates were obtained from the Department of Plant Science, University of Manitoba and AAFC-Brandon. Chemotypes of isolates were determined by *TRI3* and *TRI12* gene-specific PCR assays as detailed by Amarasinghe et al. (2019) (Supplementary Figure 1). Isolates were initially grown on 10-cm diameter potato-dextrose agar (PDA) solid growth media plates and grown to capacity (16-h light: 8-h dark) at 20°C over 2 weeks. For production of liquid media, tomatoes were cut into 1-cm^3^ cubes, and 900 g were placed in 9 L of distilled water for 3 h and then strained through cheesecloth. Fifteen grams of NaCl l^−1^ were dissolved in the filtrate and autoclaved for 20 min. One PDA culture plate from each isolate was cut into 1-cm^3^ cubes, added to 500 ml of suspension in 1 l flasks and agitated on an orbital shaker for 2 weeks under natural light at room temperature. The suspension was strained through cheesecloth, and then conidia spores were counted under 40X magnification, using a hemocytometer.

### Growth Cabinet—Experimental Design

For “Norman” and “CDC Kendall,” two seeds of same source were planted per pot (a 20-cm diameter; 30-cm depth), containing a Pro-Mix Mycorrhizae growing medium (Premier Horticulture Inc., Quakertown, PA, USA) and 15 g of slow-release granular fertilizer (18-6-8 NPK). One plant was removed from each pot, following germination. Plants were grown in a growth chamber (16-h light: 8-h dark) at an 18°C cycle. Starting at the fourth week post-seeding, plants were fertilized with 20 g l^−1^ of (20-20-20 NPK) and every 2 weeks thereafter.

Macroconidia suspensions of *F. graminearum* were diluted to 5 × 10^4^ spores ml^−1^ and mixed by chemotype with Tween20® at 0.2% vol/vol. Mock inoculation treatment included identical suspension treatment but excluded fungus. Once spikes had emerged, 75% three spikes were gently removed from the leaf sheath and entirely sprayed on either side until run-off, using an atomizer under 70 kPa of pressure. Following inoculation, spikes were covered by a translucent glycine bag for up to 96 h.

**Figure 1:**
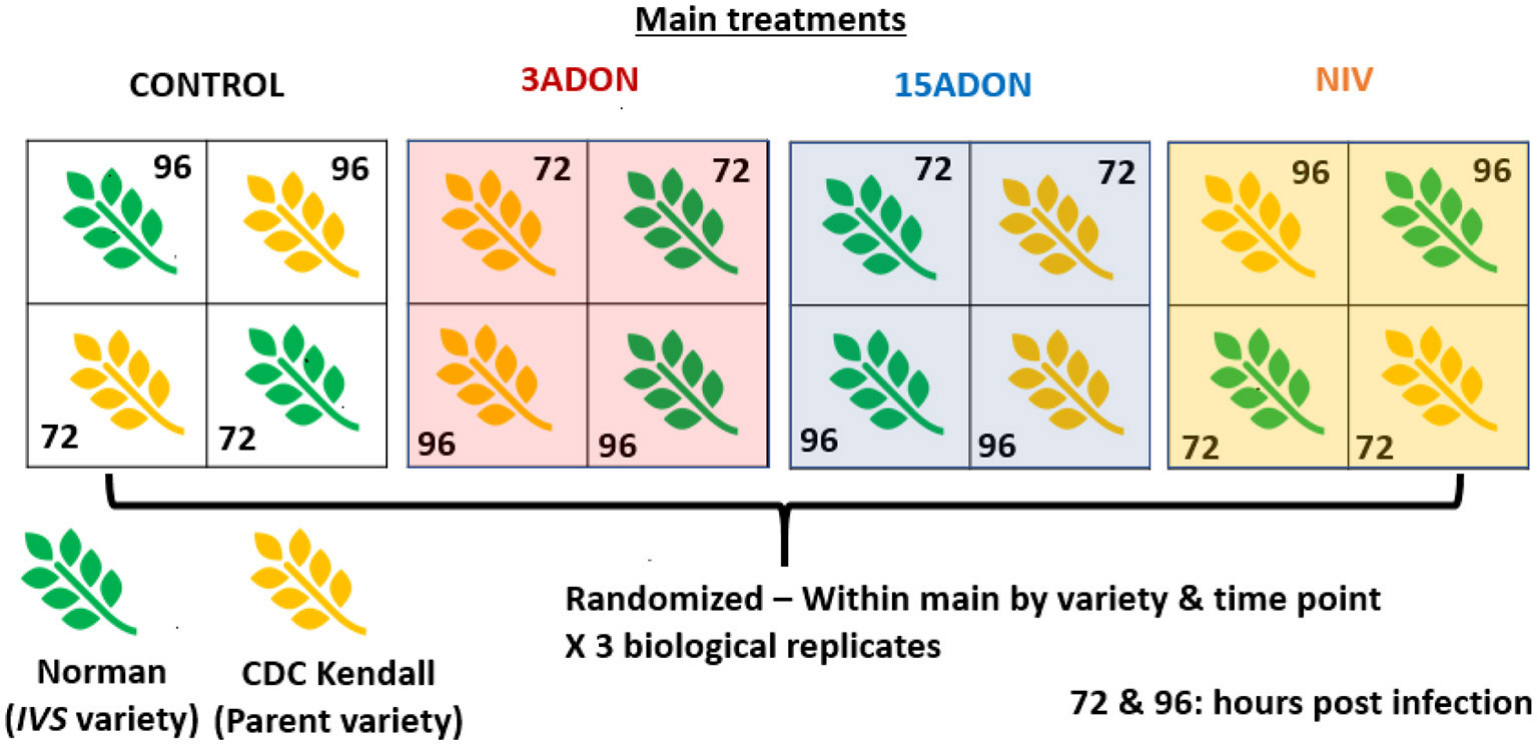
Split-plot experimental design. Main plot treatments: Control, mock control; 3ADON, 3-acetyldeoxynivalenol producer; 15ADON, 15-acetyldeoxynivalenol producer; NIV, nivalenol producer. Genotype (Norman or CDC Kendall) and sampling time point (72- and 96-h post infection) were randomized within the main plot.

A total of three biological replicates were grown per treatment and time point. Pots were arranged in the growth cabinet in a split-plot design where the treatment group (3ADON, 15ADON, NIV, or Mock inoculated) represented the main plot treatment (Figure 1). Within each main treatment, pots were completely randomized for position of variety and time point sampling (72 or 96 hpi). All plants were grown together in the same chamber. For both time points (72 or 96 hpi), spikes were cut at the peduncle at the same time of the day, wrapped in aluminum foil and flash frozen by submerging in liquid N_2_ and then held at −80°C until processed further. Remaining spikes were rated for proportion of diseased kernels at 3 weeks post-infection (diseased kernels/total kernels, Supplementary Figure 2). Plants were grown to maturity in the growth cabinet, after which spikes were harvested for mycotoxin assay. All kernels from two spikes per variety/treatment/replicate were grounded into flour together, using a handheld coffee grinder and then subsampled to 1 g. All mycotoxins were assayed at North Dakota State University by gas chromatography—mass spectrometry (GCMS) or in case of deoxynivalenol-3-O-glucoside (DON3G) by quad time of flight (QTOF).

### Statistical Analysis of FHB and Mycotoxin Content

Data were analyzed by generalized linear mixed model (GLMM) approach. Proportion of Fusarium damaged kernels (FDK) was analyzed by PROC GLIMMIX in SAS® 9.4 (SAS Institute Inc., Cary, NC, USA, SAS® 9.4, SAS Institute Inc, 2013) by using a beta distribution and logit link function to test for significant differences in the proportion FDK. Likewise, PROC GLIMMIX was used to test for differences in mycotoxin content, under lognormal distribution identity link function. Variety and chemotype treatment were considered fixed, while replicate was considered a random factor. Tukey-Kramer honestly significant difference was used to test all pairwise contrasts.

### Total RNA Extraction and Quality

Spikes were removed from the freezer, and six kernels from the mid-spike region were rapidly dissected from the rachis, pooled, and grounded to powder under liquid N_2_ *via* mortar and a pestle. Total RNA was isolated by the RNAeasy Plant Mini Kit (Qiagen, Canada) as per instructions of the manufacturer. A total of 100 mg of sample powder was used as input, followed by RLT buffer protocol. Total RNA was first checked for quality control and determining the purity of samples, using a 2100 Agilent Bioanalyzer™ (Agilent Technologies, Santa Clara, CA, USA), where RIN scores of > 7.5 were accepted.

### RNA-Seq Library Preparation and Sequencing and Quality

The cDNA libraries were constructed with Illumina TruSeq RNA v3 adaptors, using the Illumina first-strand TruSeq® mRNA library Prep Kit (Illumina, San Diego, CA) protocol as per instructions of the manufacturer. Briefly, this method purifies poly (A) mRNA from total RNA through applications of oligo (dT)-attached magnetic beads, followed by cDNA synthesis. Samples were sequenced on an Illumina HiSeq 4000 platform (100 bp paired-end) libraries. RNA-sequencing was conducted at the Centre de Services et d'Expertises Génome Québec (Montréal, QC, Canada). Fastq files for each sample were evaluated on an individual basis, using FastQC v0.11.5 software *via* visual graphic inspection (Andrews, 2010). Raw data were processed, using Trimmomatic v0.36 software (Bolger et al., 2014) to remove adaptors and trim low-quality reads.

### Sequence Alignment and Genomic Feature Evaluation

The IBSC_v2 (International Barley Sequencing Consortium) release of the barley reference genome (Mascher et al., 2017) and GTF file structural high confidence (HC) gene predictions assembly were downloaded through file transfer protocol (FTP) from Institute of Plant Genetics and Crop Plant Research (IPK) Gatersleben^2^ HISAT2 v2.0.4 software (Kim et al., 2015) was used to map clean reads to the reference genome (Supplementary Table 1).

Software package StringTie (Pertea et al., 2015) was utilized to approximate the gene expression levels in each sample from the BAM files. StringTie-merge procedure was used to input and combine lists of transcripts aligned to reference gene annotation list and newly assembled genes/transcripts. After the HC genes and new assembled genes/transcripts were merged, the expression and the coverage at all these annotations were estimated. Coverage was converted to raw reads counts by a python script within a StringTie package. In order to annotate those newly assembled genes by StringTie, the protein sequences were extracted in a fasta format from the genome reference, using gene coordinates by a pearl script. The protein fasta files were searched against a local NCBI nr database by the blastp program. A total of 52,266 genomic features were included in the gene matrix for expression analysis.

Package “edgeR” (Robinson et al., 2010) within the R statistical environment (R Core Team, 2020) was used for group comparison statistical analyses. The raw read counts of all samples were loaded as a gene matrix into “edgeR” and normalized. Factors included: genotype (“Norman” vs. “CDC Kendall”), treatment (Mock, 15ADON, 3ADON or NIV), and time (72 or 96 hpi). Generalized linear models (Appendix A) were applied both as: (i) pairwise single-factor contrasts or (ii) additive model design (a three-factor model with interaction) *via* likelihood ratio test (LRT). Identified differentially expressed genes (DEG) were examined regarding log-fold change, counts per million and significance. Genes were determined to be significantly differentially expressed if they had a log2-fold change >1 or < −1 and false discovery rate (FDR ≤ 0.05). Gene interactions of DEGs between groups were examined, using Venn diagrams, using a web-based tool.^3^

Gene ontology annotation files were downloaded from IPK Gatersleben *via* FTP^4^ Genes were assigned GO terms under categories: a biological process, cellular component, or molecular function. Significant GO terms (FDR ≤ 0.05) in each contrast were identified through GO enrichment analysis, using AgriGO v2.0 (Tian et al., 2017). ShinyGO v0.61 software (Ge et al., 2020) was used to visually evaluate relationships of DEGs in hierarchical clustering tree graphics for significant pathways where networks of GO terms were mapping, using *Arabidopsis thaliana* STRING-db v.10 with a.2 edge cut-off.

## Results

### Norman and Its Response to DON Accumulation

**Figure 2:**
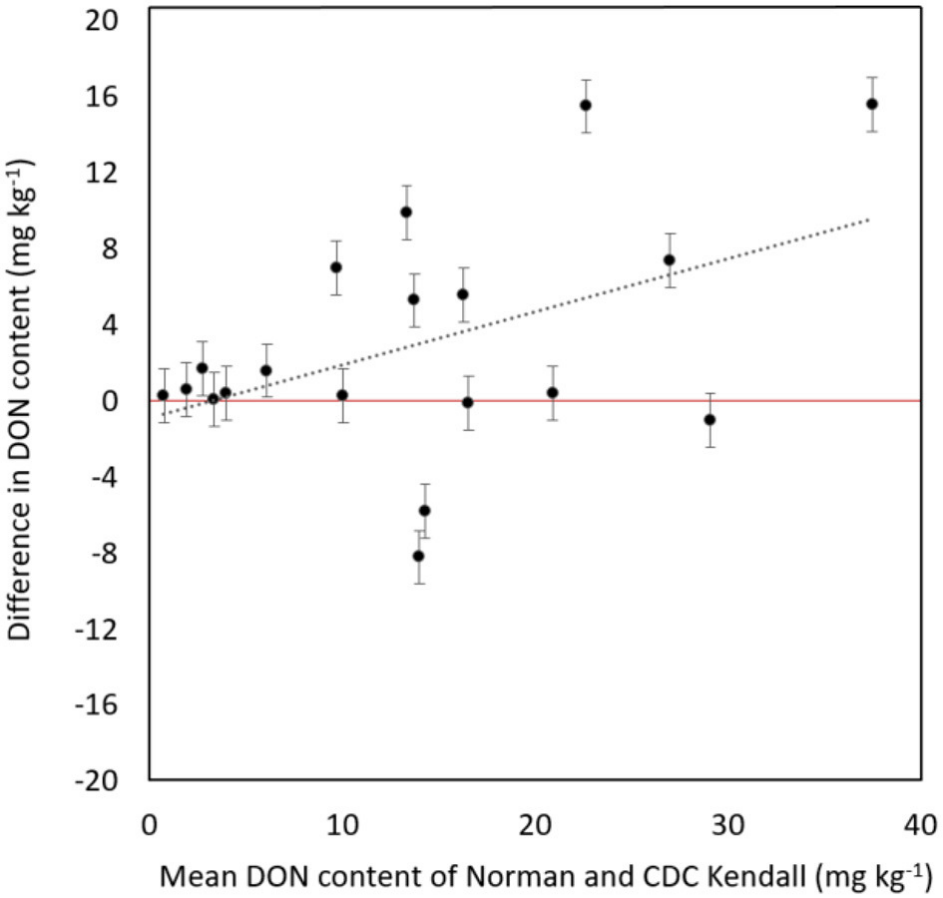
Difference in deoxynivalenol (DON) content (mg kg^−1^) of moderately resistant “Norman” in head-to-head contrast with “CDC Kendall” over 19 site years tested in an artificially inoculated disease nursery at Brandon, MB, displayed in order of mean DON content of “Norman” and “CDC Kendall.” Error bars represent standard error of difference.

“Norman” demonstrated a significant reduction of DON content in contrast with “CDC Kendall” (*P* = 0.013). “Norman” possessed lower DON content than “CDC Kendall” in most years [79% (15/19), averages not shown] of evaluation. DON levels varied considerably over years, ranging 6–45 mg kg^−1^. Reduction of DON levels in “Norman” was most prominent in epidemic years characterized with high-DON production (> 10 mg kg^−1^; Figure 2).

The proportion of FDKs was subjected to analysis of variance. No difference in visual disease symptoms was observed between “Norman” and “CDC Kendall” (*P* = 0.803, Supplementary Table 2). The proportion of FDK was significantly affected by chemotype treatment (*P* = 0.015), without interaction between variety and chemotype treatment (*P* = 0.64). The 3ADON treatment showed highest disease severity (%) at 29.8 ± 4.7, followed by NIV treatment at 18.9 ± 3.0 and 15ADON treatment at 13.3 ± 3.3 (Supplementary Figure 3). Tukey-Kramer group separation indicated that 3ADON treatment was found to display higher disease than 15ADON, but not NIV (Supplementary Table 2). Likewise, 15ADON did not differ from NIV treatment.

The 3ADON strain demonstrated significantly elevated overall mycotoxin levels in contrast with 15ADON and NIV chemotypes (Table 1). The 3ADON chemotype produced 10-fold increase of a toxin in contrast with the alternative chemotypes, where treatment was significant (*P* = 0.01, Supplementary Table 3). The 15ADON and NIV chemotype produced similar levels of mycotoxin, respectively, i.e., DON = 27.4 ± 8.6 mg kg^−1^; NIV = 39.5 ± 9.5 mg kg^−1^. Effects of variety and interaction term variety x treatment were non-significant (*P* > 0.05). The ratio of DON3G/DON trended higher in 15ADON (0.27 ± 0.1) vs. 3ADON (0.19 ± 0.1) chemotype, but this was non-significant (*P* > 0.05). The ratio did not differ between varieties within 15ADON or 3ADON treatment (*P* > 0.05). Comparison of SNP markers of 50K SNP array between “Norman” and “CDC Kendall” revealed overall a high similarity (98% SNP marker allele agreement, Supplementary Table 4). Distributions of SNP variants were examined, where regions of three chromosomes were identified: chromosome 1H: (483–512 Mb); intermittently over regions of chromosomes 4H (600–616 Mb) and 5H (621–630 Mb). Variant regions were observed on both short and long arms of chromosome 7H at multiple positions (Supplementary Figure 4).

**Table 1:** Mean ± standard error of mycotoxin content of matured barley grains for grains harvested from growth cabinet study (replication, *n* = 3).

**Variety**	**Treatment^**I**^**	**DON^**II**^**	**NIV^**II**^**	**D3G ^**III**^**	**Ratio D3G/DON**
CDC Kendall	15ADON^A^	28.1 ± 14.6	0.0 ± 0.0	5.6 ± 1.7	0.27 ± 0.1
	3ADON^B^	207.4 ± 33.0	0.5 ± 0.1	45.3 ± 13.1	0.21 ± 0.0
	NIV^A^	1.8 ± 0.7	36.5 ± 9.6	1.1 ± 0.9	0.54 ± 0.4
	Mock control	0.0 ± 0.0	0.0 ± 0.0	0.0 ± 0.0	–
Norman	15ADON^A^	26.7 ± 12.5	0.0 ± 0.0	4.6 ± 1.4	0.27 ± 0.1
	3ADON^B^	238.5 ± 31.6	0.7 ± 0.5	40.6 ± 10.2	0.17 ± 0.0
	NIV^A^	2.9 ± 2.2	42.5 ± 18.6	0.9 ± 0.5	0.50 ± 0.1
	Mock control	0.0 ± 0.0	0.0 ± 0.0	0.0 ± 0.0	–

I *Tukey grouping for treatment least squares means (α = 0.05). LS-means with the same letter are not significantly different for primary toxin—deoxynivalenol (DON) or nivalenol (NIV);*

II *mycotoxin content (mg kg^−1^) of deoxynivalenol (DON) or nivalenol (NIV) as measured by gas chromatography—mass spectrometry (GC-MS);*

III *mycotoxin content (mg kg^−1^) of deoxynivalenol-3-glucoside (D3G) measured by Quad time of flight (QTOF).*

*Statistical significance is defined by alternate letters i.e. A or B*.

### Transcriptome Differentiations

On average, the sequencing produced 64 × 10^6^ reads per library (Supplementary Figure 5). Following trimming, an average of 90% of cleaned reads per sample mapped to the IBSC_v2 reference genome (Supplementary Table 1). Graphics produced by FastQC indicated that all sample libraries passed quality standards (Supplementary Figure 6).

#### Differential Genes of CDC Kendall

**Figure 3:**
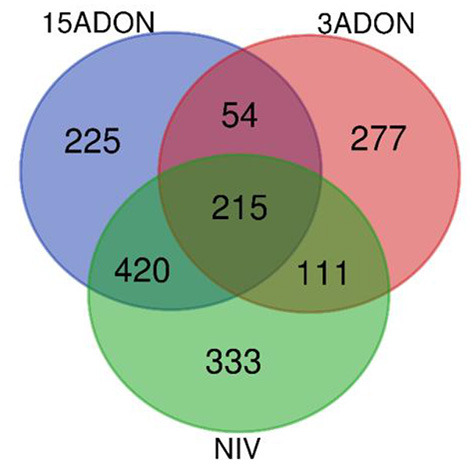
A Venn diagram of differentially expressed genes (FDR ≥ 0.05) for contrasts of *Fusarium* treatment (3ADON, 15ADON, and NIV chemotypes) vs. mock control under the additive generalized linear model (GLM).

As summarized in Table 2, overall, a total of 2,973 unique DEGs were identified through contrasts of all *Fusarium* treatments (3ADON, 15ADON, NIV) to a respective control group over both time points (72, 96 hpi). Analyses under three-factor additive GLMs for *Fusarium*-treatment groups were conducted to contrast *Fusarium*-treatment groups with the mock control group. Differentially expressed genes (1,635 total unique DEGs) were identified for the three *Fusarium* treatment groups. On examination, groups of *Fusarium*-responsive DEGs (215) with shared expression patterns were identified for all *Fusarium* treatments (Treatment intersection−15ADON⋂3ADON⋂NIV; Figure 3), indicating common response in gene groups. A selection of DEGs commonly induced in the *Fusarium* treatments was displayed (Table 3).

**Table 2:** Numbers of differentially expressed genes (FDR < 0.05) by chemotype for contrasts: CDC Kendall vs. control; Norman vs. CDC Kendall over time and 72 vs. 96 h post-infection (hpi).

		**(i) Paired, single-factor contrasts**						**(ii) Additive, interactive contrasts**		
***Fusarium_vs_control (CDC Kendall)***		**3ADON**		**15ADON**		**NIV**		***Fusarium_vs_control***		
		**72**	**96**	**72**	**96**	**72**	**96**	**3ADON**	**15ADON**	**NIV**
	Up	763	1,022	846	122	434	556	400	842	914
	Down	353	473	561	289	91	162	257	72	165
	**Total**	**1,116**	**1,495**	**1,407**	**411**	**525**	**718**	**657**	**914**	**1,079**
***Norman_vs_CDC Kendall***		**3ADON**		**15ADON**		**NIV**				
		**72**	**96**	**72**	**96**	**72**	**96**	***Norman_vs_CDC Kendall***		
	Up	77	85	964	884	116	14	53	–	–
	Down	447	892	136	435	120	12	30	–	–
	**Total**	**524**	**977**	**1100**	**1319**	**236**	**26**	**83**	**–**	**–**
***96_vs_72***		**3ADON**		**15ADON**		**NIV**				
		**CDC Kendall**	**Norman**	**CDC Kendall**	**Norman**	**CDC Kendall**	**Norman**	***96_vs_72***
	Up	478	672	1111	411	934	590	181	-	-
	Down	17	145	734	481	76	33	12	-	-
	**Total**	**495**	**817**	**1,845**	**892**	**1,010**	**623**	**193**	**-**	**-**

*Generalized linear models included: (i) paired single factor or (ii) additive, interactive, three-factor*.

**Table 3:** Transcript alterations in select upregulated genes expressed within *Fusarium graminearum* chemotype:15ADON, 3ADON, and NIV treatments contrasted with mock control identified in the additive, interactive generalized linear model.

		**15ADON**			**3ADON**			**NIV**		
**Gene**	**Annotation**	**logFC**	**logCPM**	**FDR**	**logFC**	**logCPM**	**FDR**	**logFC**	**logCPM**	**FDR**
HORVU2Hr1G104410	Ubiquitin 7	−3.2	6.1	3.1E^−19^	−3.0	6.1	2.6E^−11^	−5.0	6.1	9.7E^−31^
HORVU2Hr1G107100	Protein translation factor *SUI1* homolog 1	−2.3	6.5	1.3E^−10^	−2.0	6.5	3.9E^−06^	−3.3	6.5	4.3E^−15^
HORVU5Hr1G047150	UDP–Glycosyltransferase (*HvUGT13248*)	−2.4	6.1	9.7E^−12^	−1.5	6.1	1.5E^−04^	−2.8	6.1	1.3E^−12^
HORVU5Hr1G103770	Alpha/beta-Hydrolases protein	−2.1	5.1	1.8E^−09^	−1.1	5.1	8.1E^−03^	−2.4	5.1	1.3E^−10^
HORVU3Hr1G065320	ABC transporter B member 4	−2.0	6.5	1.4E^−08^	−1.1	6.5	2.0E^−02^	−2.5	6.5	1.4E^−10^
HORVU2Hr1G107860	Histone-lysine N–methyltransferase	−1.6	5.7	7.4E^−06^	−1.8	5.7	1.3E^−06^	−2.1	5.7	4.2E^−09^
HORVU5Hr1G056280	ABC transporter G family member 53	−2.2	6.6	2.3E^−09^	−1.5	6.6	2.0E^−04^	−1.9	6.6	3.1E^−06^
HORVU5Hr1G124650	ABC transporter G family member 48	−2.0	6.7	2.1E^−08^	−1.1	6.7	1.7E^−02^	−2.4	6.7	8.9E^−10^
HORVU3Hr1G071470	ABC transporter G family member 32	−2.1	6.2	7.9E^−10^	−1.2	6.2	8.2E^−03^	−2.2	6.2	6.8E^−09^
HORVU7Hr1G092510	ABC transporter G family member 44	−2.3	5.9	2.4E^−11^	−1.1	5.9	6.6E^−03^	−2.0	5.9	1.4E^−07^
HORVU4Hr1G026340	Transporter	−2.0	5.9	1.2E^−08^	−1.4	5.9	8.6E^−04^	−2.0	5.9	6.2E^−08^
HORVU1Hr1G047030	Cytochrome P450 protein	−2.2	6.1	5.3E^−10^	−1.1	6.1	1.8E^−02^	−2.0	6.1	2.0E^−07^
HORVU4Hr1G067450	Sugar transporter protein 7	−2.0	6.2	1.3E^−08^	−1.1	6.2	2.4E^−02^	−2.3	6.2	8.4E^−10^
HORVU5Hr1G052150	Indole-3-glycerol phosphate synthase	−2.1	6.3	5.8E^−09^	−1.1	6.3	2.0E^−02^	−2.0	6.3	1.2E^−07^
HORVU1Hr1G019750	Laccase-19	−1.8	6.6	5.8E^−07^	−1.2	6.6	1.0E^−02^	−2.0	6.3	8.6E^−08^
HORVU1Hr1G019740	Laccase-19	−1.8	6.2	3.2E^−07^	−1.2	6.2	6.8E^−03^	−2.1	6.2	2.9E^−08^
HORVU3Hr1G097860	Laccase-19	−1.7	6.3	6.7E^−06^	−1.1	6.3	1.1E^−02^	−2.0	6.3	8.6E^−08^
HORVU3Hr1G100190	Jasmonate O-methyltransferase	−1.8	5.2	1.9E^−07^	−1.1	5.2	9.5E^−03^	−2.1	5.2	2.8E^−09^
HORVU2Hr1G124930	Peroxidase superfamily protein	−1.7	5.1	8.4E^−07^	−1.1	5.1	1.0E^−02^	−1.8	5.1	1.5E^−07^
HORVU5Hr1G071140	Cellulose synthase like E1	−1.9	5.0	1.3E^−08^	−1.0	5.0	2.6E^−02^	−1.7	5.0	8.8E^−06^
HORVU5Hr1G104580	UDP-Glycosyltransferase (*HvUGT14077*)	−1.8	5.7	5.4E^−07^	−1.0	5.7	4.1E^−02^	−1.9	5.7	4.3E^−07^
HORVU5Hr1G023730	Pathogenesis-related protein *STH−21*	−1.6	5.2	1.1E^−05^	−1.1	5.2	5.5E^−03^	−1.6	5.2	1.2E^−05^
HORVU2Hr1G089440	Phenylalanine ammonia-lyase 2 (*PAL 2*)	−1.4	5.6	4.6E^−05^	−1.0	5.6	3.2E^−02^	−1.5	5.6	3.5E^−05^
HORVU3Hr1G098160	CaLB domain family protein	−1.2	5.3	1.8E^−03^	−1.1	5.3	1.3E^−02^	−1.6	5.3	2.1E^−05^
HORVU3Hr1G100350	Xylanase inhibitor	−1.2	6.3	5.2E^−03^	−1.1	6.3	8.5E^−03^	−1.1	6.3	1.2E^−02^

*logFC, log2 (fold change); logCPM, log2 (counts per million); FDR, false discovery rate, p-value adjusted by Benjamini Hochberg procedure*.

The additive interactive model identified 30 DEGs associated with “CDC Kendall” in contrast with “Norman,” which were characterized by patterns of a generalized resistance motif. “CDC Kendall” displayed predominant response in detoxification resistance (Tables 2, 4A). These genes were largely involved in defense response and intensified at 96 hpi. Genes associated with the JA/ETH pathway were elevated, including lipolytic enzyme GDSL esterase/lipase and augmented lignification response *via* lacasse 7. Several genes of oxidative-stress response were identified: GST (glutathione S-transferase), CyP450 (Cytochrome P450), and *CBSX1* (chloroplastic, redox homeostasis). Two DUF239 carboxyl-terminal proteinase-like proteins were differentially expressed (putative *DD1A*—receptor death domain protein (*Oryza sativajaponica* group)], involved in the phagocytosis of apoptotic cells). DEGs were identified associated with PR protein major pollen allergen Bet v 1 (*PR10*). Two Bowman-Birk type trypsin inhibitors were identified. Differential expression patterns were displayed for receptor genes involved in pathogen recognition.

**Table 4:** **(A)** Transcript alterations in genes differentially expressed in “CDC Kendall” identified in the additive, interactive generalized linear model. **(B)** Transcript alterations in genes differentially expressed in “Norman” identified in the additive, interactive generalized linear model.

**Gene_ID**	**Annotation [alternate species, NCBI-ProteinID]**	**logFC**	**logCPM**	**FDR**
**(A)**				
HORVU1Hr1G008360	Laccase 7	−2.1	3.4	3.3E^−09^
–	Amidase 1-like [A. tausch*ii*, XP_020159982]	−1.8	−0.2	4.6E^−07^
HORVU2Hr1G095460	Glutathione S–transferase family protein	−1.5	3.0	1.3E^−04^
HORVU2Hr1G125690	Protein of unknown function (DUF239)	−1.4	3.1	1.8E^−04^
HORVU4Hr1G016500	Tapetum determinant 1	−1.3	4.6	1.6E^−04^
HORVU3Hr1G035680	Flowering promoting factor 1	−1.3	−0.2	6.4E^−03^
HORVU1Hr1G044200	Serine carboxypeptidase–like 51	−1.2	3.7	9.1E^−04^
HORVU6Hr1G018670	Proline–rich protein, putative, expressed	−1.2	5.6	1.3E^−04^
–	Glycine/proline–rich protein [*D. hygrometricum*, KZV44779]	−1.2	−0.3	1.2E^−02^
HORVU1Hr1G038730	GDSL esterase/lipase	−1.2	6.1	1.1E^−03^
HORVU5Hr1G104800	Acyl-CoA N–acyltransferases (NAT) superfamily protein	−1.2	3.8	1.9E^−02^
HORVU4Hr1G054870	Major pollen allergen Bet v 1–F/I	−1.1	2.3	1.3E^−02^
HORVU0Hr1G005720	Pyruvate kinase family protein	−1.1	3.5	7.9E^−04^
–	Glutathione S–transferase [D. villosum, ABU56005]	−1.1	3.8	3.1E^−03^
HORVU1Hr1G021180	Glutathione S-transferase family protein	−1.1	2.7	2.7E^−02^
–	Os06g0133400, partial [*O. sativa Japonica*, BAS96003]	−1.0	3.5	6.0E^−03^
HORVU3Hr1G117370	Glutathione S-transferase family protein	−1.0	3.4	1.5E^−02^
HORVU4Hr1G080740	Protein of unknown function (DUF239)	−1.0	6.3	1.5E^−02^
HORVU2Hr1G004600	Cytochrome P450 superfamily protein	−1.0	4.6	2.3E^−02^
HORVU5Hr1G047990	invertase inhibitor [C. sativus*, XP_011660336*]	−1.0	3.0	4.6E^−02^
HORVU7Hr1G002010	Leucine–rich repeat receptor–like protein kinase protein	−0.9	2.1	4.2E^−02^
–	Synaptic vesicle 2–related protein [T. urartu*, EMS56043*]	−0.9	2.8	4.5E^−02^
–	Os05g0341100 [O. sativa Japonica*, BAS93469*]	−0.9	4.6	2.2E^−02^
HORVU6Hr1G014600	Bowman-Birk type trypsin inhibitor	−0.9	3.9	2.7E^−02^
HORVU0Hr1G025520	Leucine-rich repeat receptor–like protein kinase protein	−0.9	3.8	4.1E^−02^
HORVU7Hr1G002930	Disease resistance protein (CC-NBS-LRR class)	−0.9	3.6	2.1E^−02^
HORVU1Hr1G068300	Somatic embryogenesis receptor kinase 1 (*SERK1*)	−0.9	4.7	4.6E^−02^
–	CBSX1 [*A. tauschii*, XP_020147453]	−0.8	3.2	4.0E^−02^
–	Pectinesterase inhibitor [*H. vulgare*, ACR61378]	−0.8	6.5	4.7E^−02^
HORVU0Hr1G000760	Proteinase inhibitor [*T. aestivum*, AAS49905]	−0.8	4.3	4.9E^−02^
**(B)**				
HORVU3Hr1G075620	tRNA pseudouridine synthase D	0.8	3.9	4.2E^−02^
HORVU6Hr1G000680	Thionin 2.1	1.0	3.1	2.5E^−02^
HORVU6Hr1G000780	Thionin 2.1	1.0	4.5	7.1E^−03^
HORVU1Hr1G070310	Aldose reductase	1.0	6.2	3.5E^−03^
HORVU2Hr1G071890	Desiccation-related protein PCC13-62	1.1	5.1	4.8E^−04^
–	Protein *FAR1*-Related [*A. tauschii*, XP_020188798]	1.1	0.0	1.4E^−02^
HORVU1Hr1G087380	Protein of unknown function (DUF679)	1.1	4.0	4.5E^−04^
HORVU6Hr1G013580	F-box domain containing protein	1.2	3.4	3.3E^−04^
–	Zinc finger CCHC domain protein 8 [*T. urartu*, EMS62713]	1.2	1.6	1.1E^−02^
HORVU4Hr1G006120	Splicing factor Prp18 family protein	1.2	1.5	3.4E^−02^
HORVU7Hr1G025460	Homeobox-leucine zipper protein ROC8	1.2	4.0	1.7E^−04^
–	Ribosomal protein L34Ae [*C. cardunculus*, KVI00286]	1.2	2.5	4.9E^−04^
HORVU7Hr1G082040	Late embryogenesis abundant protein D-34	1.2	3.1	1.7E^−04^
HORVU4Hr1G059260	Heat shock 70 kDa protein 3	1.3	6.1	3.2E^−05^
HORVU7Hr1G025490	Homeobox-leucine zipper protein *ROC8*	1.3	1.7	1.4E^−03^
HORVU1Hr1G079280	Late embryogenesis abundant protein 76	1.3	1.0	1.5E^−03^
HORVU5Hr1G091920	Copine family protein 2	1.3	1.2	1.5E^−03^
HORVU5Hr1G094680	Late embryogenesis abundant protein D-34	1.3	4.1	3.5E^−05^
HORVU4Hr1G005690	Antimicrobial peptides-like [*A. tauschii*, XP_020185846]	1.3	3.5	1.1E^−04^
HORVU7Hr1G025450	Homeodomain leucine zipper family IV protein	1.3	1.4	1.3E^−03^
HORVU7Hr1G064800	Unknown protein—Os07g0120700 [*O. sativa*, BAF20695]	1.4	0.2	4.9E^−04^
HORVU7Hr1G025480	Homeodomain leucine zipper family IV protein	1.4	0.7	1.1E^−03^
HORVU4Hr1G087780	Bifunctional inhibitor/LTP/seed storage 2S albumin	1.4	4.0	9.3E^−07^
HORVU2Hr1G077710	22.0 kDa class IV heat shock protein	1.4	3.9	3.4E^−05^
HORVU5Hr1G112540	Protein synthesis inhibitor II	1.5	6.9	6.6E^−07^
HORVU1Hr1G059900	Late embryogenesis abundant protein D-19	1.6	6.0	5.4E^−07^
HORVU7Hr1G083750	Protein kinase superfamily protein	1.8	2.3	3.3E^−09^
HORVU7Hr1G010680	Acid phosphatase 1	2.3	6.9	1.9E^−10^
HORVU7Hr1G006260	Avenin-like a3	2.5	3.0	1.3E^−17^
HORVU4Hr1G007680	Eukaryotic aspartyl protease family protein	2.7	2.5	1.3E^−14^
HORVU2Hr1G116500	Avenin-like a4	3.0	2.3	1.1E^−26^
HORVU7Hr1G011180	Retinol dehydrogenase 14	3.3	1.4	7.7E^−25^

*logFC, log2 (fold change); logCPM, log2 (counts per million); FDR, false discovery rate, p-value adjusted by Benjamini-Hochberg procedure*.

#### Differential Genes of Norman

A total of 53 DEGs associated with “Norman” were identified through the additive interactive model (Tables 2, 4B). This group of genes, which were strongly expressed in a pattern earlier in “Norman” at 72 hpi and, particularly, in the 15ADON treatment. DEGs, including numerous abscisic acid (ABA) stress-responsive proteins; late embryogenesis abundance (LEA), heat-shock, desiccation related; Aldose reductase (HORVU1Hr1G070310). Multiple antifungal defenses of PR proteins were induced earlier in “Norman” at 72 hpi, including thionin 2.1 (*PR13*), defensin-like protein (*PR12*), bifunctional inhibitor/lipid-transfer protein/seed storage 2S albumin superfamily protein (*PR14*), protein synthesis inhibitor II (ribosome-inactivating—rRNA N-glycosidase), HORVU4Hr1G005690 (antimicrobial peptide *A. tauschii* homolog), and glucan endo-1,3-beta-glucosidase GI (*PR2*). Differential response was also observed in a xylanase inhibitor.

#### Norman vs. CDC Kendall Control

A total of 473 and 580 DEGs were observed at 72 and 96 hps (hours post sprayed), respectively. Several genes appeared to be constitutively expressed earlier in “Norman,” including peroxidase class III BP1 (*HvPrx5*); ascorbate peroxidase (HORVU2Hr1G009940), cathepsin B-like cysteine, subtilisin-like protease and chymotrypsin inhibitor, serpin (Z7), C-hordein, vicilin (HORVU5Hr1G104630) and germin-like protein (HORVU3Hr1G011990, *PR15*), LEA protein *HvA1* heat-shock protein, and disease-resistance proteins.

#### Differential Genes Between Norman and CDC Kendall by Treatment and Time Point

Paired contrasts from single-factor GLM were conducted between the two genotypes within *Fusarium*-treatments and at both time points. Total DEGs identified between “Norman” and “CDC Kendall” ranged from only 26 for NIV at 96 hpi to 1,319 for 15ADON at 96 hpi (Table 2). Under the three-factor additive GLM model, an overall total of 83 DEGs were identified between varieties (Table 2). Variance associated with a genotype was minimal compared to *Fusarium* treatments and/or time, as might be predicted for varieties sharing 98% of their genetic code (Supplementary Table 4, Supplementary Figure 4). However, a complex resistance response occurred where resistance factors were expressed in either variety, with differences observed over treatment and time.

#### Contrast 72 vs. 96-h Post-infection

Patterns of temporal gene expression were observed in both varieties. Paired contrasts (72 vs. 96 hpi) for single-factor GLM were conducted within “Norman” and “CDC Kendall.” DEGs where identified within each treatment group (Table 2). Dominant patterns of elevated gene expressions over time were observed in 3ADON and more so within NIV treatments, while more balance was observed in the 15ADON treatment. Under the three-factor additive GLM for 72 vs. 96-treatment contrast patterns indicated intensification of gene response over time, where a total of 193 DEGs were identified which were principally upregulated (Table 2). A selection of DEGs, which increased over time, is displayed in Table 5.

**Table 5:** Transcript alterations in select upregulated genes from 72- to 96-h post-infection identified in the additive, interactive generalized linear model.

**Gene**	**Annotation**	**logFC**	**logCPM**	**FDR**
HORVU2Hr1G104410	Ubiquitin 7	−2.1	6.1	1.0E^−09^
HORVU2Hr1G107100	Protein translation factor *SUI1* homolog 1	−2.0	6.5	1.0E^−09^
HORVU0Hr1G038820	60S acidic ribosomal protein P0	−1.8	4.4	4.3E^−07^
HORVU3Hr1G006940	16.9 kDa class I heat shock protein 2	−1.7	4.0	4.0E^−09^
HORVU0Hr1G007340	Progesterone 5 beta reductase	−1.4	4.5	2.6E^−06^
HORVU1Hr1G046630	Valine—tRNA ligase	−1.4	4.5	6.0E^−06^
HORVU2Hr1G104390	Ubiquitin 11	−1.4	4.2	1.1E^−05^
HORVU7Hr1G096690	Chromosome 3B, genomic scaffold, cultivar Chinese Spring	−1.3	5.5	8.2E^−06^
HORVU5Hr1G056280	ABC transporter G family member 53	−1.2	6.6	1.1E^−04^
HORVU5Hr1G096930	Cytochrome P450 superfamily protein	−1.2	4.7	2.1E^−05^
HORVU1Hr1G063390	AAA-ATPase 1	−1.1	5.4	6.3E^−05^
HORVU5Hr1G047150	UDP–Glycosyltransferase (*HvUGT13248*)	−1.1	6.1	7.6E^−04^
HORVU2Hr1G032330	Sulfotransferase 17	−1.1	4.9	2.7E^−04^
HORVU6Hr1G034570	Receptor kinase 2	−1.1	4.1	2.0E^−03^
HORVU5Hr1G115100	GRAM domain/ABA-responsive protein–related	−1.0	4.4	4.1E^−04^
HORVU7Hr1G002820	Alpha dioxygenase	−1.0	4.8	2.7E^−04^
HORVU4Hr1G065800	Inactive poly [ADP-ribose] polymerase *RCD1*	−1.0	5.1	1.3E^−03^
HORVU4Hr1G063350	Heat shock protein 21	−1.0	5.1	5.4E^−04^
HORVU3Hr1G052880	ATP-dependent zinc metalloprotease *FtsH 2*	−1.0	4.7	1.8E^−03^
HORVU2Hr1G094230	1-aminocyclopropane-1-carboxylate synthase 11	−1.0	4.4	1.3E^−03^
HORVU6Hr1G089560	U-box domain-containing protein	−1.0	4.4	1.0E^−03^
HORVU1Hr1G038700	Cysteine–tRNA ligase	−1.0	4.8	1.6E^−03^
HORVU6Hr1G089590	U–box domain-containing protein	−1.0	4.6	1.8E^−03^
HORVU2Hr1G012340	UDP-Glycosyltransferase superfamily protein	−1.0	4.3	7.7E^−03^
HORVU3Hr1G053350	ABC transporter G family member 36	−1.0	6.0	2.3E^−03^
HORVU5Hr1G106850	ABC transporter G family member 48	−0.9	6.9	7.3E^−03^
HORVU3Hr1G075040	L-tyrosine decarboxylase	−0.9	4.2	2.7E^−03^
HORVU3Hr1G084260	Leucine-rich repeat (LRR) family protein	−0.9	4.8	4.8E^−03^
HORVU5Hr1G112930	C2H2-like zinc finger protein	−0.9	4.8	4.0E^−03^
HORVU5Hr1G020900	GRAS family transcription factor	−0.9	4.7	2.3E^−03^
HORVU5Hr1G042030	Cytochrome P450 superfamily protein	−0.9	5.1	6.1E^−03^
HORVU2Hr1G107860	histone-lysine N–methyltransferase ATXR2	−0.9	5.7	7.4E^−03^
–	NF–X1–type zinc finger protein [*A. tauschii*, XP_020150167]	−0.9	4.9	5.5E^−03^
HORVU1Hr1G010210	Bidirectional sugar transporter N3	−0.9	4.8	4.0E^−03^
HORVU4Hr1G005500	50S ribosomal protein L18	−0.9	4.4	1.6E^−02^
HORVU5Hr1G096940	Cytochrome P450 superfamily protein	−0.9	6.5	1.2E^−02^
HORVU6Hr1G063480	Cysteine synthase D1	−0.9	4.4	1.8E^−02^
HORVU4Hr1G026340	Transporter	−0.9	5.9	1.4E^−02^
–	Fusarium resistance orphan protein [*T. aestivum*, AKZ18235]	−0.9	4.3	3.7E^−02^
HORVU2Hr1G027470	Cytochrome P450 superfamily protein	−0.9	5.9	1.1E^−02^
HORVU4Hr1G053130	Calmodulin 3	−0.9	5.1	1.2E^−02^

*logFC, log2 (fold change); logCPM, log2 (counts per million); FDR, false discovery rate, p-value adjusted by Benjamini-Hochberg procedure; annotation as per Barlex, IPK-Gatersleben*.

**Figure 4:**
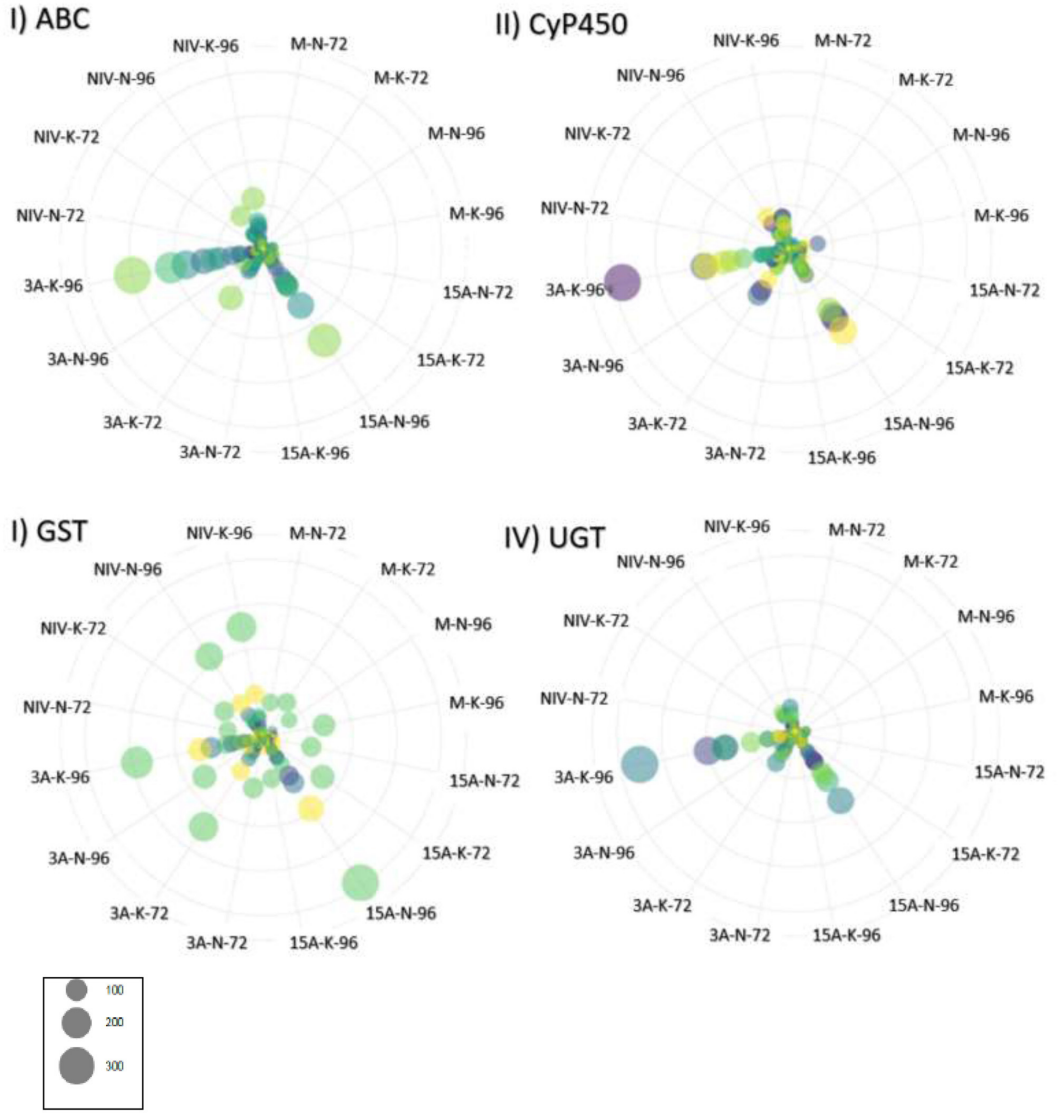
Gene expression for gene families associated with resistance to deoxynivalenol (DON) expressed in transcripts per million (TPM). Genes are identified by color, and circle size corresponds to TPM. **(I)** ABC, ATP-binding cassette transporter (*n* = 24 genes); **(II)** CyP450, cytochrome P450 superfamily protein (*n* = 42 genes); **(III)** GST, glutathione S-transferase (16 genes); **(IV)** UGT, UDP-Glycosyltransferase superfamily proteins (36 genes). M, mock control; 3ADON, 3-acetyldeoxynivalenol; 15ADON, 15-acetyldeoxynivalenol; NIV, nivalenol. 72-and 96-h post-infection.

Elevated patterns of upregulation were observed at 96 hpi for genes associated with *Fusarium* reaction, including trichothecene detoxification and oxidative stress response. Extreme elevations of gene expressions were seen in ABCs (ATP-binding cassette transporters), CyP450s (Cytochrome P450s), GSTs (glutathione S-transferases), sugar transporters, and UGTs (UDP-glucuronosyltransferases) (Figures 4I–IV). Upregulation was observed in peroxidases, LEAs, LOXs, and subtilisin-like protease (HORVU7Hr1G024930). Promoter sequences of DEGs identified gibberellin-regulated transcription factor *Gamyb* of the *Myb/SANT* family were a significantly enriched motif over time (FDR = 0.04). Intensive expression of genes occurred in “CDC Kendall” under the 3ADON treatment, where many DON-responsive genes were elevated by an order of magnitude at 96 hpi. Pathogen recognition increased over time marked by increased expressions in LRR proteins, MAPKKKs, protein kinases, calmodulin Ca^2+^ binding protein, and numerous WRKY transcription factors, including WRKY's 33, 50, and 75. Alternations in transcriptional patterns were evidenced by heightened expression of ubiquitination, a translation initiation factor, splicing factor Prp18 family, and ribosomal proteins. Many genes, initially lowly expressed in the NIV treatment at 72 hpi, demonstrated a significant upsurge in both varieties by 96 hpi.

#### Unique Resistance Patterns Were Observed in Norman in 15ADON Treatment at 72 hpi

DEGs of “Norman” expressed at 72 hpi within the 15ADON treatment were associated with nutrient reservoir activity of storage proteins and defense response. Genes of this kind were not transcribed in “CDC Kendall” at 72 hpi but were expressed later at 96 hpi. Defense response to pathogen cell wall digestive enzymes was seen in xylanase inhibitors. Defense in “Norman” was expressed *via* multiple protease inhibitors: Serine protease inhibitors: serpin (Z4 and Z7 families), subtilisin-chymotrypsin inhibitors (CI-1A and CI-1B), alpha-amylase inhibitor 28, and metallocarboxypeptidase inhibitor. Plant toxins expressed, such as protein synthesis inhibitor II (HORVU5Hr1G112540), rRNA N-glycosidase (ribosome-inactivating, ricin A-chain activity). Multiple RNases were observed, which could act to repress fungal effectors. Expression was observed in pathogen responsive proteins with antifungal activity, including defensin-like protein, thionin 2.1, and chitin-binding metabolite agglutinin isolectin 3. Auxin-related genes were enhanced in “Norman” at 72 hpi, as indicated by upregulation of multiple tryptophan aminotransferase-related protein 1 (*TAR1*) genes. Many senescence-associated proteins showed elevated response. Also observed was high expression in ribose-binding protein 1 homolog.

Lower expression was seen in “Norman” for gibberellin receptor *GID1* (HORVU1Hr1G060810, gibberellin insensitive dwarf), which binds to gibberellins to facilitate GID1-DELLA complexing and subsequent degradation *via* ubiquitination. DELLA protein scarecrow-like 5 (HORVU3Hr1G058100, *Sln1*-like) was differentially expressed earlier in “Norman.” Numerous ABA stress-related genes were induced, including LEA proteins [HORVU1Hr1G058400, HORVU1Hr1G079290 (*HVA1*), HORVU2Hr1G099870, HORVU4Hr1G051780, HORVU4Hr1G073120]; laccase 7 (HORVU2Hr1G002440); Dehydrin 6 (HORVU4Hr1G010750, HORVU6Hr1G084070); Dehydrogenase/reductase SDR 4 (HORVU1Hr1G018140, retinol dehydrogenase); heat shock protein [HORVU3Hr1G020500 (17.6 kDa class II), HORVU4Hr1G059260 (70 kDa protein 3)]; thioredoxin 2 (HORVU6Hr1G078960, mitochrondrial ROS homeostasis); peroxidase superfamily protein (HORVU4Hr1G022280). Significant increase was seen in nutrient storage-related proteins like cupin seed storage proteins, including vicilin-like antimicrobial peptides (HORVU4Hr1G050210, HORVU4Hr1G070970, HORVU5Hr1G104630), globulin-2 (HORVU4Hr1G002800), triticin, and germin-like proteins (HORVU5Hr1G010880, HORVU6Hr1G089570, *PR15*) (Supplementary Figure 7).

#### 3ADON Treatment at 72 hpi

Under infection by the more virulent 3ADON isolate group, “Norman” and “CDC Kendall” also displayed differential response patterns. Numerous resistance-responsive genes demonstrated higher expression in “CDC Kendall” than “Norman.” Elevated gene expression in “CDC Kendall” in phenylalanine ammonia-lyase (*PAL*) genes. Elevations were observed in UGT 74F1 (HORVU2Hr1G012280), which may conjugate glucose to salicylic acid (SA), thereby inactivating it. The Octadecanoid pathway was prominent along with many JA-related metabolite genes: Lipoxygenases (*LOX2, LOX3*, and *LOX6*), Lipase/lipoxygenase—PLAT/LH2 proteins, Patalin-like protein 5 (HORVU4Hr1G075070), Allene oxide synthase (*AOS*), and Jasmonate O-methyltransferase (*MeJA*). Extremely high levels of 12-oxophytodienoate reductase (*OPR2*) was observed in “CDC Kendall” at 72 hpi in contrast with “Norman.”

Tryptophan metabolism was indicated by anthranilate synthase component 1 (HORVU4Hr1G061120) and tryptophan synthase beta chain 1 (HORVU7Hr1G073460). Defense-related genes were congruently expressed: Thaumatin-like pathogenesis-related proteins (*PR5*), cysteine-rich venom protein, and allergen V5/Tpx-1-related protein (*PRB1-2*). Also, defense response was seen in chitinases (*PR3*), wound-induced protein (HORVU3Hr1G113120, *wheatwin-2*), and pathogenesis-related protein (*PR10*). Cell wall fortification was evident in upregulated lignification *via* laccase-19, peroxidases (HORVU2Hr1G026450, HORVU3Hr1G074960), cellulose synthase-like D3 (HORVU1Hr1G022900), and extension (hydroxyproline-rich glycoproteins). Detoxification response was enhanced by ABC transporters and UGT gene *HvUGT13248*.

While robust gene expressions were observed in “CDC Kendall,” similar defenses also occurred in “Norman” in the 3ADON treatment at 72 hpi where elevated genes expressions unique to “Norman” included chromosome 3B, genomic scaffold, cultivar Chinese spring (HORVU7Hr1G006250, LEA protein wheat homolog), CyP450 (HORVU4Hr1G083930, 86B1 family involved in very long chain fatty acids omega-hydroxylation), glucan endo-1,3-beta-glucosidase GI (*PR2*), and peroxidase *BP1* (*HvPrx5*). “Norman” demonstrated relatively higher expression in specific JA-induced proteins: 60 kDa—rRNA N-glycosidase; 32.7 kDa dirigent proteins and defensin-like protein (HORVU4Hr1G082400, *PR12*). Stress response was observed in upregulation of calcium-dependent lipid-binding protein (HORVU3Hr1G098160, CaLB domain) and response in homolog Fusarium resistance orphan protein, *TaFROG* (TraesCS4A02G20190).

Low expression of defense genes was observed under NIV treatment at 72 hpi. Some resistance-related genes were found to show elevated expression in “Norman” at 72 hpi, including peroxidase *BP1* (*HvPrx5*), lipid-transfer proteins (*PR14*), protein synthesis inhibitor II, vicilin antimicrobial peptides, homeobox-leucine zipper protein ROC, GDSL esterase/lipase, late embryogenesis abundant protein D-19 (HORVU1Hr1G059900), *PHV A1*, and several heat shock proteins.

#### Norman Displayed Alterations in Responsive Patterns in 15ADON Treatment at 96 hpi

At 96 hpi, a significant change in resistance pattern was observed in “Norman” from what was seen initially at 72 hpi. As seen in “CDC Kendall” 3ADON treatment, a secondary phenylpropanoid metabolite pathway was induced at 96 hpi, seen in upregulation of phenylalanine synthase (HORVU5Hr1G052150) and *PAL*, shikimate kinase 1, 4-coumarate-CoA ligase (*4CL*), dihydroflavonol 4-reductase (HORVU2Hr1G033610, HORVU6Hr1G089460, *DFRA*). Detoxification genes were induced: ABC, CyP450, GST, and UGT (Figures 4I–IV). Oxidative-stress response was seen under elevations in alternative oxidase 1a (HORVU2Hr1G101980 *AOX1A*), peroxidases, progesterone 5 beta reductase (HORVU0Hr1G007340), germin-like proteins (oxalate oxidase, *PR15*), and laccases. Elevations occurred in resistance-responsive DEGs included Thaumatin-like pathogenesis-related protein 1 (HORVU7Hr1G122120), cysteine-rich venom protein (HORVU7Hr1G033530, *PRB1-2*), major pollen allergen Bet v 1-F/I (HORVU4Hr1G054870, *PR10*). Protease inhibitor families (Bowman-Birk type and chymotrypsin inhibitors) and xylanase inhibitor were expressed within “Norman.”

While “Norman” at 96 hpi followed the patterns of the generalized resistance motif as seen in “CDC Kendall,” several resistance-responsive genes were enhanced in “Norman,” including defense-related PR proteins: *PRB1-2, PR3, PR5, PR10, PR15*, plant basic secretory (BSP) protein (*PR17*), and pathogenesis-related protein (HORVU3Hr1G111600). Other elevated defensive response was observed in GST (*GSTU6*), stress responsive protein (HORVU2Hr1G017420), wound-induced protein (HORVU3Hr1G113120, *wheatwin*), *MYC2* transcription factor (HORVU7Hr1G038900), and NBS-LRR resistance proteins. “Norman” expressed influential genes of cell function: Indole-2-monooxygenase-like (HORVU3Hr1G011850, benzoxazinoid synthesis), *WIR1A* proteins (HORVU5Hr1G010130, HORVU6Hr1G088880, cell wall structure), and water supply *via* aquaporin PIP2-4 (HORVU6Hr1G05893). Nitric oxide (NO)-producing nitrate reductase 1 genes (HORVU6Hr1G003300, HORVU6Hr1G079700) were also induced and preferentially in “Norman.” Cathepsin B-like cysteine proteinases (HORVU2Hr1G036930, HORVU5Hr1G095580, HORVU5Hr1G061770), boron transporter 1 (HORVU5Hr1G022140, *HvBOR1*), copper ion-binding protein (HORVU7Hr1G036180), and metacaspase 1 (HORVU3Hr1G095700) genes indicated programmed cell death response in “Norman.”

#### DON Avoidance Mechanisms Were Pronounced in CDC Kendall in 3ADON Treatment at 96 hpi

Detoxification genes initially expressed in “CDC Kendall” at 72 hpi continued to rise to extremely high expression levels at the 96 hpi time point under 3ADON treatment, in dramatic contrast to “Norman,” which was more restrained (Figures 4I–IV). The phenylpropanoid pathway was greatly induced with genes *PAL* and *4CL*. Dihydroflavonol 4-reductase (*DFRA*) and other flavonoid genes were differentially expressed, such as chalcone synthase (HORVU2Hr1G004170, *CHS*) and chalcone-flavanone isomerase (HORVU5Hr1G046480, *CHI*). JA and ETH hormone signatures were highly apparent. Pathogen recognition was evident through common expression of many receptors, modifiers, and signaling cascades, including leucine-rich repeat receptor (LRR) kinases [HORVU3Hr1G084260 (*BAK1*), HORVU3Hr1G104940, and HORVU1Hr1G068380, *SERK2*], together with modifiers of *BAK1* (MOB) kinase activator-like 1A and 1B (HORVU0Hr1G018730, HORVU6Hr1G009410, and HORVU0Hr1G037740). Also evidenced were many genes of signaling cascade molecules: Numerous protein kinase superfamily proteins (MAPKKK), calmodulin-binding protein (HORVU0Hr1G002930), calcium-dependent protein kinase (*HvCPK28*), and calcium-transporting ATPase protein (HORVU1Hr1G076950). Bowman-Birk type trypsin inhibitors and *Fusarium*-specific orphan gene *TaFROG* were induced.

Heightened expression was observed in “CDC Kendall” for genes associated with respiratory burst (HORVU0Hr1G013380, HORVU4Hr1G081670, HORVU5Hr1G078630, NADPH oxidase homolog D, *HvRbohD*). Arginine decarboxylase was upregulated, which is involved in biosynthesis of polyamine spermidine. Polyamine oxidase was also activated, which is involved in H_2_0_2_ production in the apoplast. Upregulation was seen in multiple 2-oxoglutarate and Fe (II)-dependent oxygenase superfamily proteins (HORVU6Hr1G088430, HORVU2Hr1G004280, HORVU6Hr1G088440, *2OGO*). Stress-responsive genes were upregulated, including NAC domain protein (HORVU5Hr1G111590, *SNAC1*) and peroxidase. Numerous CyP450 superfamily proteins that were highly expressed involved in redox reactions. CyP450s (HORVU1Hr1G080680, CyP450 94B3 and HORVU6Hr1G001520, CyP450 94C1) are involved in modification of jasmonoyl-L-isoleucine (JA-Ile) and attenuation of JA-response. Ubiquitinization (HORVU7Hr1G109650, HORVU2Hr1G104410) and negative regulatory factor of DON-resistance, *NF-X1*-type zinc finger protein increased over time. Extremely high expression was observed in UGT proteins *HvUGT13248* and *HvUGT14077* vs. control (log_2_FC = 9.6 and 6.4, respectively). Negative regulation of cell death was apparent through a display of *BON* association protein 2 (HORVU5Hr1G080820, *BAP2*) and *MLO*-like protein 1 (HORVU0Hr1G008830) and inactive poly (ADP-ribose) polymerase *RCD1* (HORVU4Hr1G065800, *radical-induced cell death 1*). A boron efflux transporter (*HvBOR1*) was activated, where boron is a mitochondrial stabilizer and inhibitor of apoptosis. Programmed cell death-associated protein metacaspase-1 was expressed in “Norman” but was highly suppressed in “CDC Kendall” at this time.

While detoxification resistance was engaged, patterns of gene expression in “Norman” under 3ADON at 96 hpi showed some similarities to those observed in “Norman” at 72 hpi in 15ADON treatment. Vicilins (HORVU4Hr1G070970, HORVU5Hr1G104630), Serpins (Z7), subtilisin-chymotrypsin inhibitors (CI-1A and CI-1B), and B hordeins storage proteins were all elevated in “Norman.” Likewise, amine oxidase 1 (HORVU2Hr1G012710), *TAR1* proteins, xylanase inhibitor (HORVU2Hr1G043890), LEA-like protein (HORVU1Hr1G058400, *NHL13*), NAC transcription factor (HORVU6Hr1G01938, *HvNAM-1*), defensin-like protein (*PR12*), and peroxidase BP1 (*HVPrx5*). A serine/threonine-protein kinase protein was expressed in “Norman” at these stages (HORVU1Hr1G064110, *PBL7* homolog).

#### NIV Treatment at 96 hpi

**Figure 5:**
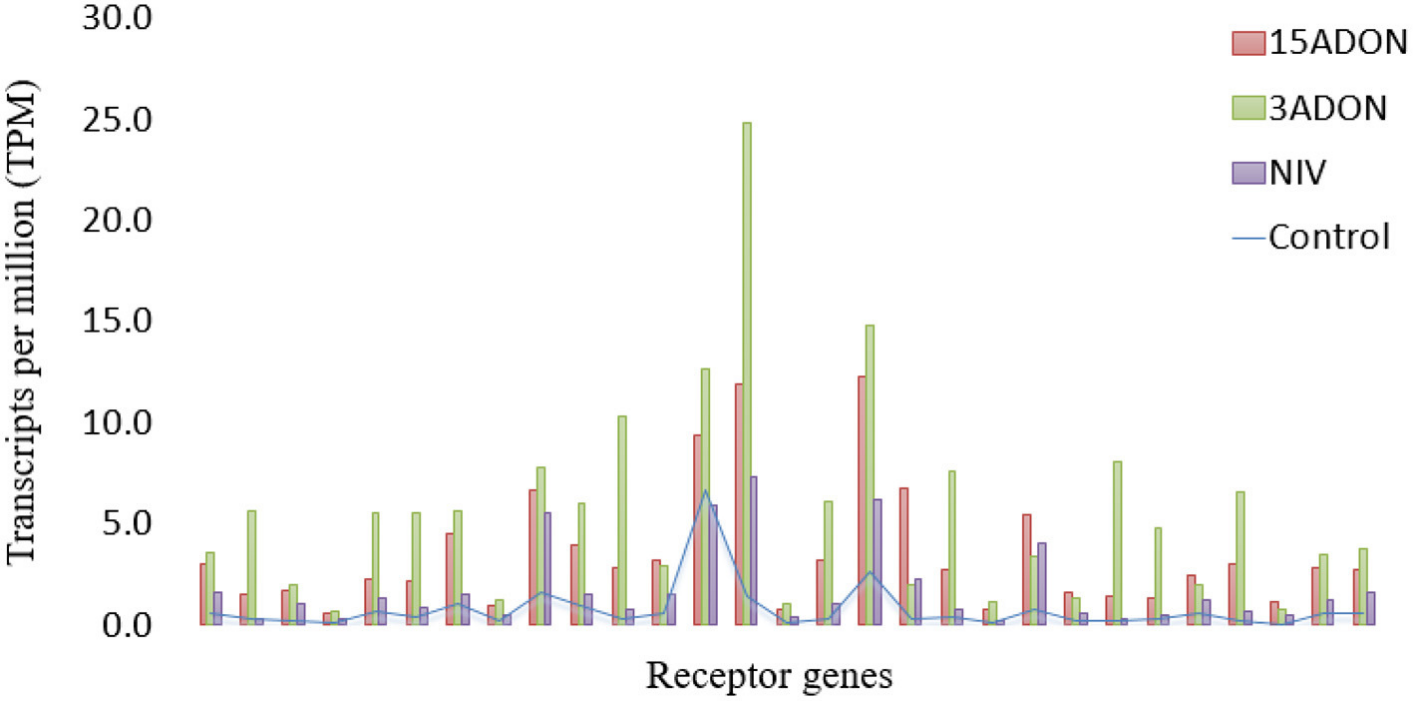
Differentially expressed genes of receptor classification (*n* = 29) for “CDC Kendall” in treatment groups (15ADON, 3ADON, and NIV) at 72 hpi vs. mock control expressed in transcripts per million (TPM).

Receptors and signaling genes were slow to be induced in the NIV treatment at 72 hpi, in contrast to DON-producers (Figure 5). However, by 96 hpi, the NIV chemotype demonstrated an upsurge in expression, where gene families were similar to those seen in DON-producing treatments. The varieties showed very similar gene expressions at 96 hpi; however, germin-like (*PR15*), aspartic proteinase nepenthesin and peroxidase *BP1* (*HvPrx5*) protein expressions were higher in “Norman.” *GST*s displayed a higher induction than *UGT*s under the NIV vs. DON treatments (Figures 4, III and IV).

#### Gene Ontology (GO) Enrichment in Fusarium-Treatment vs. Control

**Figure 6:**
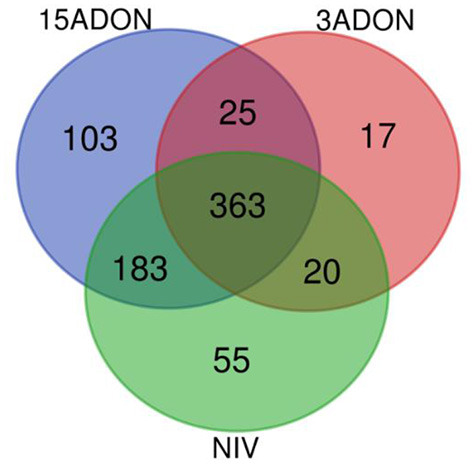
A Venn diagram of enriched categories of GO terms (FDR ≥ 0.05) associated with differentially expressed genes in additive generalized linear model (GLM) contrast of mock control and Fusarium treatment (3ADON, 15ADON, and NIV chemotypes).

An overrepresentation analysis was used to evaluate DEGs from treatment contrasts to mock control to identify GO (Gene Ontology) terms enriched for the *Fusarium*-barley interaction. In contrast to the mock control group, the *Fusarium*-treatments displayed upregulation in a suite of genes. Groups of statistically significant GO terms (FDR ≥ 0.05) were analyzed for all treatment groups (15ADON, 3ADON, and NIV). GO terms between treatments were compared for intersection and similarity of expression patterns. Gene processes displayed common enrichment of mechanisms among the groups (Figure 6).

GO-enrichment portrayed increased unification of host response of coordinated mechanisms. Approximately half (47%) of all enriched terms was identified as common to all *Fusarium* treatment groups, including biological process terms: “L-phenylalanine metabolic/catabolic process”; “ammonium transmembrane transport”; “phenylpropanoid metabolic process”; “lignin metabolic process”; “oxidation-reduction process”; “organonitrogen compound catabolic process”; “oxoacid metabolic process”; “carboxylic acid metabolic/catabolic process”; “cellular amino acid catabolic process” (GO:0009063); “aminoacyl-tRNA ligase activity” (GO:0004812), tryptophan biosynthetic process (GO:0000162), and indolalkylamine (GO:0042430; GO:0046219), which was evidenced by strong upregulation of anthranilate synthase component 1 (HORVU4Hr1G061120), tryptophan synthase. Pathogen response was perceived in “response to biotic stimulus” (GO:0009607) and “establishment of localization” (GO:0051234).

**Figure 7:**
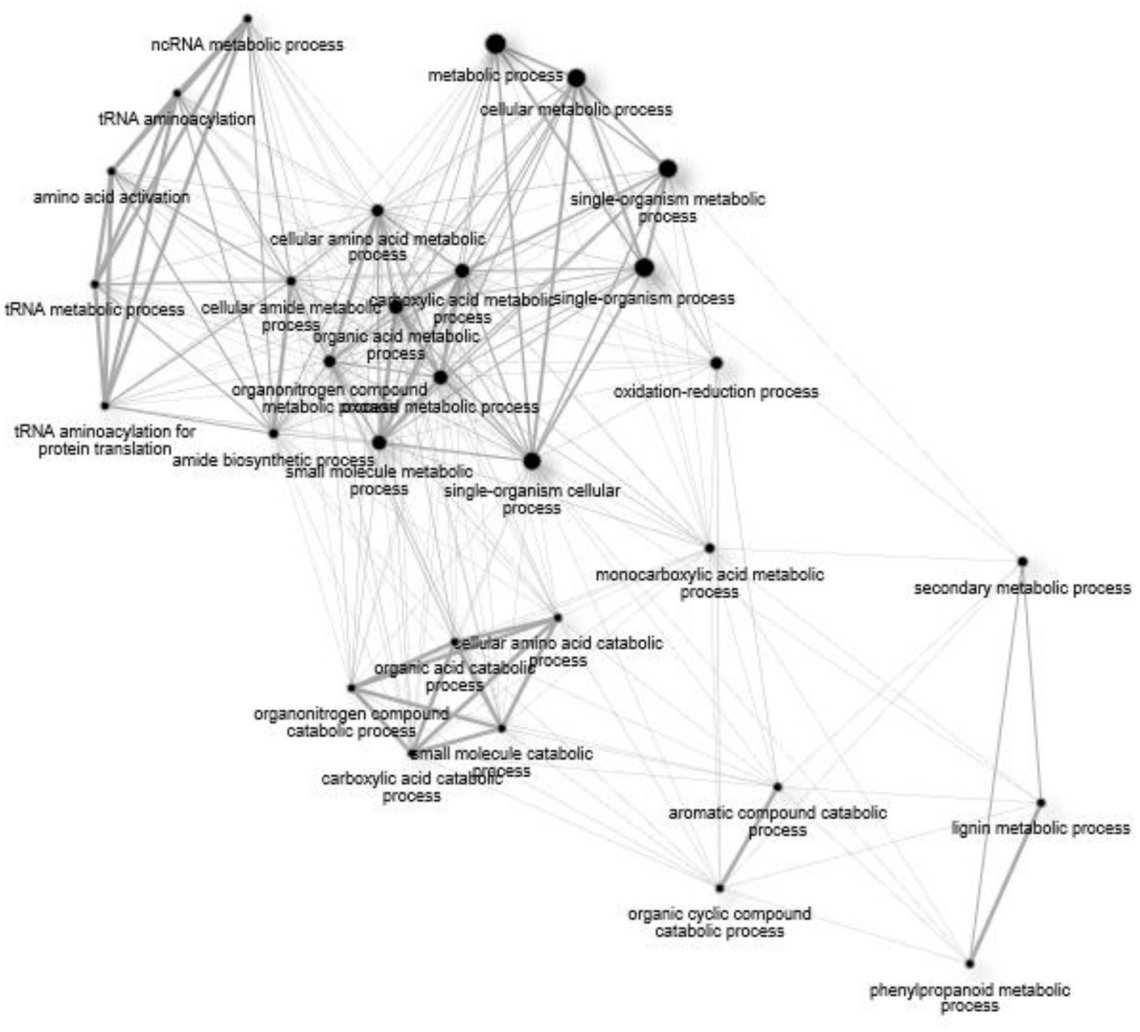
A network of significant (FDR > 0.05) Gene Ontology (GO) terms for biological processes common to all treatment groups (15ADON, 3ADON, and NIV).

Figure 7 displays a network assembly of GO terms common to the *Fusarium* treatments. Non-coding RNA metabolism generates alterations in amino acid composition, which influence a metabolic process. Transcriptional reprogramming of *Fusarium*-response was directed at the level of amino acid metabolism through activation of aminoacyl-tRNA synthetase for particular amino acids (Ala, Cys, Leu, Lys, Phe, Pro, Val). Indole-3-glycerol phosphate synthase (HORVU5Hr1G052150) was upregulated, which participates in aromatic amino acid biosynthesis. Single-organism processes stimulate oxidation-reduction processes, which, in turn, influence aromatic compound catabolism. Processes together lead to production of secondary metabolism of phenylpropanoids and lignification response. This resistance pattern occurred in both varieties.

### GO Enrichment in 72 vs. 96 hpi

The additive GLM, identified significant (FDR < 0.05) enrichment of biological processes over all treatments. Enrichment was observed with gene groups associated with elevated functions at 96 hpi. Changes in cellular reprogramming were apparent through augmentations in “protein ubiquitination” (GO:0016567) “ubiquitin-protein transferase activity” (GO:0004842) were observed arising from expression of multiple U-box domain-containing proteins (HORVU1Hr1G069990, HORVU6Hr1G089560, HORVU6Hr1G089590, HORVU7Hr1G040790). “Anion transport” was identified as a process-enriched term, including boron transporter 1 (HORVU5Hr1G022140) and high-affinity nitrate transporter 3.1 (HORVU6Hr1G053710, NAR2.3). “Sequence-specific DNA binding” (GO:0043565) was amplified, including genes encoding multiple WRKY transcription factors of the JA signaling pathway. Functional enrichment was observed in calcium ion binding (GO:0005509) in calcium-transporting ATPase (HORVU1Hr1G076950). “ATPase activity” (GO:0016887) and “ATPase activity,” coupled to movement of substances (GO:0043492) associated with elevations of ABC transporter activities.

Pairwise contrasts also identified common enrichment in processes over time, including a “phenylpropanoid metabolic process” (GO:0009698) and a “lignin metabolic process” (GO:0009808), such as laccases and a “cell wall macromolecule metabolic process” (GO:0044036). “Defense response” (GO:0006952), “alternative oxidase activity (GO:0009916). Regulatory functions were enriched, including “regulation of biosynthetic processes” (GO:0009889), including genes rRNA N-glycosidase, protein synthesis inhibitor II, and quinone; “regulation of gene expression” (GO:0010468) enrichments was seen in NAC, WKRY, ERF, bZIP families. Enhanced molecular function was seen in “peptidase inhibitor activity” (GO:0030414), nutrient reservoir activity (GO:0045735) seen in increase of storage proteins. Temporal patterns of enrichment demonstrated similar progressions within individual *Fusarium*-treatment groups.

### GO Enrichment in “Norman” vs. “CDC Kendall”

The most significant category of gene enrichment for the biological process was “defense response” (Table 6). Enrichment was also seen in response to “biotic stimulus,” “response to stress,” and “response to stimulus.” Enriched categories of DGEs displayed negative biological regulatory processes. Molecular functions were enriched in elements of pathogenic repression. Enriched elements indicated that differences in host-pathogen interaction response occur within the “extracellular region.”

**Table 6:** Gene Ontology (GO) terms of “Norman” vs. “CDC Kendall” contrast identified in the additive, interactive generalized linear model.

**GO code**	**Type**	**Term**	**FDR**
GO:0006952	P	Defense response	4.9E^−20^
GO:0051248	P	Negative regulation of protein metabolic process	4.1E^−10^
GO:0034249	P	Negative regulation of cellular amide metabolic process	4.1E^−10^
GO:0032269	P	Negative regulation of cellular protein metabolic process	4.1E^−10^
GO:0017148	P	Negative regulation of translation	4.1E^−10^
GO:0009607	P	Response to biotic stimulus	1.9E^−8^
GO:0010608	P	Posttranscriptional regulation of gene expression	2.5E^−8^
GO:0034248	P	Regulation of cellular amide metabolic process	2.5E^−8^
GO:0006417	P	Regulation of translation	2.5E^−8^
GO:0031327	P	Negative regulation of cellular biosynthetic process	4.1E^−7^
GO:0009890	P	Negative regulation of biosynthetic process	4.1E^−7^
GO:2000113	P	Negative regulation of cellular macromolecule biosynthetic process	4.1E^−7^
GO:0010558	P	Negative regulation of macromolecule biosynthetic process	4.1E^−7^
GO:0031324	P	Negative regulation of cellular metabolic process	5.5E^−7^
GO:0051172	P	Negative regulation of nitrogen compound metabolic process	5.5E^−7^
GO:0032268	P	Regulation of cellular protein metabolic process	6.3E^−6^
GO:0006950	P	Response to stress	8.7E^−6^
GO:0051246	P	Regulation of protein metabolic process	1.7E^−5^
GO:0010629	P	Negative regulation of gene expression	3.3E^−5^
GO:0009892	P	Negative regulation of metabolic process	4.7E^−5^
GO:0010605	P	Negative regulation of macromolecule metabolic process	4.7E^−5^
GO:0048523	P	Negative regulation of cellular process	5.5E^−5^
GO:0048519	P	Negative regulation of biological process	6.7E^−4^
GO:0050896	P	Response to stimulus	4.6E^−3^
GO:0044712	P	Single-organism catabolic process	3.4E^−2^
GO:0030597	F	RNA glycosylase activity	2.2E^−10^
GO:0030598	F	rRNA N-glycosylase activity	2.2E^−10^
GO:0004867	F	Serine-type endopeptidase inhibitor activity	1.4E^−9^
GO:0030414	F	Peptidase inhibitor activity	5.4E^−8^
GO:0004866	F	Endopeptidase inhibitor activity	5.4E^−8^
GO:0061135	F	Endopeptidase regulator activity	5.4E^−8^
GO:0061134	F	Peptidase regulator activity	5.4E^−8^
GO:0016799	F	Hydrolase activity, hydrolyzing N-glycosyl compounds	3.9E^−7^
GO:0004857	F	Enzyme inhibitor activity	2.5E^−6^
GO:0030234	F	Enzyme regulator activity	1.2E^−4^
GO:0098772	F	Molecular function regulator	2.7E^−4^
GO:0045735	F	Nutrient reservoir activity	9.9E^−3^
GO:0005576	C	Extracellular region	5.0E^−10^

*P, biological process; F, molecular function; C, cellular component; FDR, false discovery rate, p-value adjusted by Benjamini-Hochberg procedure*.

### The Differential Gene Associated With Oxidative Stress Reduction

*Fusarium*-treatments clearly demonstrated a sign of oxidative stress through activation of multiple genes-encoding proteins with antioxidant genes with oxidoreductase activity included: numerous peroxidases, L-ascorbate oxidase, thioredoxin 2, ferredoxin 3, FAD-binding Berberine family protein, quinone, 2-oxoglutarate, and Fe (II)-dependent oxygenase superfamily, and lignin-associated laccase-19. Oxidative stress was apparent under *Fusarium*-treatments through expression of ubiquinol oxidase-alternative oxidases, *AOX1A* (HORVU2Hr1G101920), *AOX2* (HORVU2Hr1G101920, HORVU2Hr1G101990), and external alternative NADPH-ubiquinone oxidoreductase B3 (HORVU7Hr1G073050). These genes that provide alternative oxygen reduction mechanisms within electron transport chain and mitigate problems of excess ROS for maintaining growth. Likewise, induction of ATP-dependent zinc metalloprotease *FtsH 2* (HORVU3Hr1G052880) indicated potential photo-damage of thylakoid membranes. Such genes were expressed in excess in “CDC Kendall,” indicating higher oxidative stress. GO terms for molecular function were enriched in activities associated with oxidative stress and detoxification: “alternative oxidase activity” (GO:0009916); “oxidoreductase activity” (GO:0016491); “oxidoreductase activity, acting on diphenols and related substances as donors, oxygen as acceptor” (GO:0016682); “iron ion binding” (GO:0005506); “heme binding” (GO:0020037). Enrichment was seen in all treatment groups in response to an oxidative-stressed state of a host, following *Fusarium*-infection.

## Discussion

We document in the current study that 3ADON chemotypes created more disease and much higher mycotoxin in barley than 15ADON or NIV chemotypes. DON is a virulence factor contributing to primary infection in barley (type I resistance) but, in contrast to wheat, does not contribute to increased spread from point of initial infection (Jansen et al., 2005). Resistance to spread from point of infection (type II resistance) is robust in barley, where even susceptible barleys can adequately impede internal growth through the rachis, owing to strong detoxification mechanisms. As seen in previous studies of six-row barley (Boddu et al., 2007; Gardiner et al., 2010; Huang et al., 2016), two-row barley in the current study demonstrates a coordinated defense response through a suite of DON-induced genes. Elevated co-expression of detoxification genes was observed in the 3ADON treatment and predominantly in “CDC Kendall,” including CyP450's, which modify harmful molecules and/or facilitate oxidative reactions of phenylpropanoid production. Glutathione S-transferase (GST) and UGTs conjugate toxins to create glutathione or glucose moieties. ABC transporters facilitate transport of xenobiotics out of a cell or into vacuoles. Detoxification resistance was common to both varieties and was increasingly expressed to 96 hpi.

Both “Norman” (moderately resistant variety) and “CDC Kendall” (intermediate variety) displayed strong detoxification responsive against DON. UDP-glucuronosyltransferase (UGT) genes like *HvUGT13248*, which converts DON to DON3G through addition of a glucose molecule, are very well-characterized (Schweiger et al., 2010) and are essential components of type II resistance. Barley UGTs like *HvUGT13248* are very effective, where enhanced type II resistance was attained by insertion of this transgene in wheat (Li et al., 2015). Virus-induced gene silencing of *HvUGT-10W1*, an allelic variant of *HvUGT14077*, resulted in compromise type II resistance, allowing spread through the spike (Xing et al., 2017). DON conjugation transforms mycotoxins to less-toxic forms but also facilitates mobilization to apoplast/vacuoles (Audenaert et al., 2014) or binding to cell wall constituents (Zhou et al., 2008). While the enhanced rate of DON-glycosylation is associated with wheat resistance (Kluger et al., 2015), the ratio of DON3G/DON in barley was similar in mature grains of differentially resistant varieties (Tucker et al., 2019). This suggests that resistance in barley is likely not enhanced by elevated UGT gene expression. While DON conjugation effectively lowers overall toxin content, conjugates are also undesirable as they can readily be hydrolyzed to the principal toxin by common gut bacteria (Berthiller et al., 2011).

“Norman” displayed a more conservative response to DON under challenge by more aggressive 3ADON-producing strains under pathogen progression. Overstated detoxification response in “CDC Kendall” suggests a host-pathogen interaction of increased mycotoxin content. In environments where pathogens may demonstrate proclivities for toxin production, reliance on DON resistances involving avoidance mechanisms may not be adequate. Generation of reactive oxygen species (ROS) through an oxidative burst is observed under first stages of cereal host defense response to *F. graminearum* (Taheri, 2018). As seen by Huang et al. (2016), a large number of receptor kinase genes were invoked in the current study linked with pathogen recognition and resultant signaling cascades. ROS molecules associated with the oxidative burst operate directly as protective agents, trigger programmed cell death (PCD), and/or signal pathogenesis-related (PR) protein induction (Lamb and Dixon, 1997). DON elicits H_2_O_2_ production in cereals, which, in juxtaposition, may trigger antimicrobial defense but may stimulate cell death, which ultimately benefits necrotrophic growth (Desmond et al., 2008). *Fusarium graminearum* responds reciprocally to ROS through stress-responsive generation of DON (Ponts et al., 2007), thereby establishing a positive feedback loop, leading potentially to toxin amplification. However, the more-virulent 3ADON form demonstrated abilities to increase infection rates and mycotoxin production in either variety. The increased frequency of occurrence of these more virulent forms poses a serious threat to barley production. Focus on incorporating robust resistance to initial infection within DON-based resistance may be an increasingly important resistance mechanism for challenges to more-virulent strains.

Phenylalanine ammonia-lyase (*PAL*) genes were highly expressed in both varieties herein and the study conducted by Huang et al. (2016). *PAL* catalyzes a deamination reaction, converting L-phenylalanine to trans-cinnamic acid. It represents a shift from primary to secondary metabolic function and the first step of the biosynthetic process of the polyphenol compounds, such as flavonoids, phenylpropanoids, and lignin. *PAL* is an important component of the basal resistance mechanism of barley associated with *MYB* transcription factors. Mass spectrometry applications have confirmed common occurrence of resistance-related compounds in *Fusarium*-infected cereals, where flavonoid phenylpropanoids are predominant (reviewed by Gauthier et al., 2015). Lignin formed through polymerized phenolic precursors contributes to resistance *via* fortification of physical barriers but is also important as a precursor of JA biosynthesis (Chamarthi et al., 2014). Boutigny et al. (2008) implicate phenolic compounds as DON suppressors through quenching oxidative stress. However, defenses of this nature are generally slow and may be outpaced by more virulent pathogens. Phenolic acids generally inhibit *F. graminearum* growth; however, compounds with higher antioxidant properties may also stimulate increased toxins *via* stress response (Ponts et al., 2011).

Jamonic acid/ ETH pathways were induced by *Fusarium* in either variety, where response increased over time. Kumaraswamy et al. (2012) demonstrated JA involvement in transcriptional response to DON in wild type to *TRI5*- contrast. “CDC Kendall” and “Norman” were characterized with elevated JA-response in later treatment, while *PR* expressions were generally more pronounced in “Norman.” JA-response under study was associated with lignification, detoxification mechanisms, and elevated expressions genes, such as chitinases (*PR3*) and thaumatin-like (*PR5*) proteins. Geddes et al. (2008) demonstrated these *PR* proteins and oxidative burst/stress response occurred both in moderately resistant and intermediately resistant two-row barley varieties infected by *F. graminearum*. In the current study, DEGs were primarily associated with JA vs. salicylic acid (SA) expression; however, SA-signatures may also have disappeared by this time point as seen by Huang et al. (2016). Hormones are important coordinating factors in quantitative disease traits and are principally involved in response to a pathogen, where SA is typically associated with resistance to biotrophs vs. JA/ETH, which is common in necrotrophic response (Glazebrook, 2005). Basal resistance, activated early in the defense response of cereals, is associated with SA accumulation (Makandar et al., 2012; Hao et al., 2018; Wang et al., 2018). Resistance associated with JA/ETH signaling pathways was also implicated in several cereal studies (Li and Yen, 2008; Gottwald et al., 2012; Makandar et al., 2012; Qi et al., 2016; Wang et al., 2018). Antagonism between hormones is documented in cereal response to *F. graminearum*, where host compatibility is complicated by a hemi-biotrophic lifestyle. Resistance occurs in orderly sequence, where resistance coordination may be important for fully effective resistance (Ding et al., 2011; Makandar et al., 2012).

We observed antagonism between stress-responsive ABA and growth-related GA phytohormones. In cereals, ABA is a phytohormone associated with the onset of stress-related metabolites, anti-oxidative activation, stomatal closure, and lowered photosynthetic efficiencies (Gietler et al., 2020). GA insensitive dwarf 1 (*GID1*) receptor interacts with GA and DELLA to form a protein complex (Murase et al., 2008), followed by marking of DELLAs *via* F-box proteins for ubiquitinization and degradation. Degradation of GA-repressive DELLA-encoding *Slender1* (HORVU3Hr1G058100, *Sln1*) gene permits the GA-signaling pathway. Mutations in the DELLA domain required for interaction with *GID1 and* GA-growth response result in a semi-dwarf phenotype, which is susceptible to biotrophic pathogens but resistant to necrotrophic pathogens and *F. graminearum* (Saville et al., 2012). De Bruyne et al. (2014) discuss the role of DELLAs in providing resistance to necrotrophs through induction of compounds, which alleviate oxidative stress, repress cell wall loosening, and compete with *JAZ* molecules, allowing *MYC2* to bind G-box and release JA-responsive genes. “Norman” at 72 hpi demonstrated an elevated level of elevated ABA-responsive genes and senescence-related proteins, while GA-signatures (*Gamyb*) dominated the defense response of DON-detoxification at 96 hpi, particularly under the 3ADON treatment. Petti et al. (2010) also observed downregulation of GA-receptor and elevated DELLA expression and disease control of *F. culmorum*, following induction of induced systemic resistance (ISR) *via* priming of *Pseudomonas fluorescence*. Interplay of ABA-GA phytohormones may play a role in modulating the biotrophic-necrotrophic transition. This antagonism is a basis of trophic divergence in a diversified host-pathogen interaction, which may result in both resistance or susceptibility under species-specific circumstance (Huang et al., 2020).

Oxidative stress visible in all *Fusarium*-treatments likely is the result of elevated ROS production. “Norman” demonstrated a reduced state of oxidative stress relative to “CDC Kendall.” Abiotic stress response of ABA phytohormones may overlap with biotic pathways that may occur *via* stimulus of water-stress and electrolyte leakages, arising from cell wall damage (López et al., 2008). The host needs effective means to control oxidative stress to minimize cell damage. Late embryogenesis abundant (LEA) proteins are a large group of hydrophilic, stress-induced proteins termed as such due to fact that they occur at the onset of seed desiccation. LEA proteins were associated with rachides of *Fusarium*-resistant wheat lines (Liu et al., 2019). A wheat trans-gene (*TdLEA3*) expressed in *A. thaliana* demonstrated (Koubaa and Brini, 2020) increased stress tolerance and oxidative stress reduction through protection of ROS-scavenging genes. Numerous LEA genes were expressed early and at higher levels in “Norman.” While peroxidase expression was common to both varieties in *Fusarium*-treatments, barley peroxidase class III *BP1* (*HvPrx5*) gene was highly expressed, particularly in “Norman.” This barley peroxidase is uniquely endosperm-specific (Rasmussen et al., 1991) and was also identified as an important defensive gene by Petti et al. (2010), who speculated it to contribute to increasing cross-linkages in cell walls.

Expression patterns in “Norman” differed from “CDC Kendall” for genes, which may impede successful colonization through pronounced antimicrobial expression. Multiple xylanase inhibitors were expressed in “Norman,” which combat *Fusarium* hydrolytic enzymes, which degrade cell walls. While *F. graminearum* employs toxins as virulence factors, it is also equipped with a comprehensive secretome, including over 600 proteins (Brown et al., 2012). Secreted proteins include cell wall-degrading enzymes, which contribute as virulence factors through enhanced penetration of physical barriers. Some xylanase inhibitors are also believed to possess antifungal function, with homologous similarities to chitinase III (*PR8*) proteins (Wu et al., 2013). “Norman” displayed elevated rRNA N-glycosidase (ribosome-inactivating protein, Ricin A-chain), which could either function as an antifungal toxin or facilitate cell suicide. In the current study, antimicrobial peptides were identified earlier and with elevated expression in “Norman,” including Thionin 2.1 (HORVU6Hr1G000680, HORVU6Hr1G000780), non-specific lipid-transfer proteins (ns-LTP2) (HORVU4Hr1G087780, *PR14*) and a defensin-like protein (HORVU4Hr1G082400, *PR12*). Hao et al. (2020) demonstrated a thionin gene in *A. thaliana*-conferred resistance to *F. graminrearum* but also raised *PR*-genes through SA and JA/ET hormone stimulation. Such cysteine-rich antimicrobial peptides were differentially expressed in moderately resistant wheat variety “Dream” and recognized as key resistance factors (Gottwald et al., 2012).

“Norman” displayed an intensified expression of proteases, including serpins, (serine protease inhibitors, which act on both as trypsin and chymotrypsin), subtilisin-chymotrypsin inhibitor, alpha-amylase inhibitors, and metallocarboxypeptidase inhibitors. Alternatively, Bowman-Birk-type inhibitors (trypsin inhibitors) were dominant in “CDC Kendall,” particularly at 96 hpi. Petti et al. (2010) also documented subtilisin/chymotrypisins and serpin proteases as key actors in barley response to *F. graminearum*. Serpins play an essential role in regulation of programmed cell death (Dickman and Fluhr, 2013) and may represent important mechanisms for control of *Fusarium*-induced death response. Cell death may occur as a defense mechanism but also is induced by DON. Pekkarinen et al. (2007) conducted kinetic studies of protease kinds and found chymotrypsin/subtilisin inhibitor 2 (CI-2) to be more efficient than Bowman-Birk-type trypsin inhibitors, while both were considered capable of binding *Fusarium* proteinases which may inhibit barley *PR* defense proteins. Alpha-amylase inhibitor, which was highly pronounced in “Norman,” was identified by Zantinge et al. (2010) as a protein found of higher abundance in FHB-resistant barleys. Success of more aggressive strains of *F. graminearum* is associated with increased protease levels vs. molecular diversification (Fabre et al., 2019), so stronger expressions may be important for control of more-virulent strains. Eggert and Pawelzik (2011) observed very little degradation of storage proteins under high FHB infection of barley, implying strong resistance mechanisms protect the endosperm. “Norman” was enriched for vicilin storage proteins, which carry antimicrobial properties.

NIV-producers incited the most similar response between the two varieties under evaluation, implying that the resistance in “Norman” may be DON specific. NIV producers do not share the same mycotoxin-induced stress response to H_2_O_2_ as DON producers, as they have higher adaptation to oxidative stress (Ponts et al., 2009). While not as aggressive as the 3ADON-producers, the NIV producers demonstrated equal ability to infect barley and accumulate mycotoxins as the 15ADON producers. While NIV may be less toxic to plants, it is more toxic to humans (Ferrigo et al., 2016). Climate changes, which may extend northern latitudes of fungi, coupled with sexual recombinant cycles and wind-dispersed ascospores, supply conceivable threats of NIV producers to northern barley production regions of North America. Given its pathogenic capabilities on barley and potential for increased toxicity, such NIV forms should be monitored through chemotype surveillance.

In the current study, we investigated genetic variation of a moderately resistant *IVS* variety in contrast to its parent variety, where differential response was observed. As seen in many other genetic studies resistance to DON, accumulation was complicated, where different forms of resistance occurred. While resolution limitations exist due to the fact that whole seeds were ground together, this study identifies global transcriptional alterations involved in the host defense response within developing grains. Resistance genes differentially expressed in “Norman” were observed in divergent relationship to generalized resistance mechanisms associated with DON avoidance. Resistances in “Norman” may impede *Fusarium* growth through antimicrobial compounds and reduce DON-inducing oxidative stress *via* antioxidants. Limitations imposed by more-virulent 3ADON forms capable of producing higher levels of DON remain challenging, where barley breeders will need to continue incorporating resistance to maintain DON content below the very low limits set by industry. Overlap was seen in resistances in either variety, implying that control of response may shape an outcome. The study has identified many genomic features, which may contribute to the barley resistance response. New technologies, such as microlaser dissection and digital droplet PCR that combine histological and transcriptomic methodologies, can be applied to further investigate features identified in this study at the single-cell level to illuminate host–pathogen interactions within specific organelles.

## Permission to Reuse and Copyright

Her Majesty the Queen in Right of Canada as represented by the Minister of Agriculture and Agri-Food Canada 2021; This article is an open access article distributed under the terms and conditions of the Creative Commons Attribution (CC BY) license.

## Data Availability Statement

The data discussed in this publication have been deposited in NCBI's Gene Expression Omnibus (Edgar et al., 2002) and are accessible through GEO Series accession number GSE174081 (https://www.ncbi.nlm.nih.gov/geo/query/acc.cgi?acc=GSE174081).

## Author Contributions

JT, AB, and WF conceived and designed the experiments. JT and WL conducted field analysis. JT grew a plant and fungus, conducted inoculations, performed the RNA isolation, and wrote the manuscript. JT, SM, WX, and ZY analyzed the data. CH conducted microarray analysis. SS conducted mycotoxin assays. AB and WF held supervisory roles. JT, AB, WF, and WX revised the manuscript. All authors read and approved the final manuscript.

## Conflict of Interest

The authors declare that the research was conducted in the absence of any commercial or financial relationships that could be construed as a potential conflict of interest.

## Publisher's Note

All claims expressed in this article are solely those of the authors and do not necessarily represent those of their affiliated organizations, or those of the publisher, the editors and the reviewers. Any product that may be evaluated in this article, or claim that may be made by its manufacturer, is not guaranteed or endorsed by the publisher.

## Footnotes

^1^https://beerandbrewing.com/dictionary/l4mIxiZeCz/).

^2^160404_barley_pseudomolecules_masked.fasta; Hv_IBSC_PGSB_r1_HighConf.gtf.gz; https://webblast.ipk-gatersleben.de/barley_ibsc/downloads/.

^3^https://bioinformatics.psb.ugent.be/webtools/Venn/.

^4^Hv_IBSC_PGSB_r1_functionalDescriptions_HighConf_Gos.tab; https://doi.ipk-gatersleben.de/DOI/57ce5c4c-3584-48fe-a33f-2544a06e555f/a27d49c1-10a3-42ce-9d43-25cea920a5f4/2.

## Appendix A

A list of 15 analyses conducted for differential gene expression and overrepresentation of Gene Ontology (GO) terms.

***Paired, single-factor GLM:***

**Fusarium treatment contrasts (2x** ***n* =** **3 samples):**

1. 15ADON Fusarium vs. mock control for CDC Kendall (72 and 96 hpi)
2. 3ADON Fusarium vs. mock control for CDC Kendall (72 and 96 hpi)
3. NIV Fusarium vs. mock control for CDC Kendall (72 and 96 hpi)

**Varietal contrasts (2 x** ***n* =** **3 samples):**

4. CDC Kendall vs. Norman, within mock control (72 and 96 hpi)
5. CDC Kendall vs. Norman, within 15ADON Fusarium (72 and 96 hpi)
6. CDC Kendall vs. Norman, within 3ADON Fusarium (72 and 96 hpi)
7. CDC Kendall vs. Norman, within NIV Fusarium (72 and 96 hpi)

**Time contrasts (2x** ***n* =** **3 samples):**

8. 72 vs. 96 hpi, within 15ADON Fusarium (Norman and CDC Kendall)
9. 72 vs. 96 hpi, within 3ADON Fusarium (Norman and CDC Kendall)
10. 72 vs. 96 hpi, within NIV Fusarium (Norman and CDC Kendall)

***Additive, three-factor GLM:***

11. 72 vs. 96 hpi (2 x *n* = 24 samples)
12. Norman vs. CDC Kendall (2 x *n* = 24 samples)
13. 15ADON (2 x *n* = 12 samples; 15ADON Fusarium vs. mock control)
14. 3ADON (2 x *n* = 12 samples; 3ADON Fusarium vs. mock control)
15. NIV (2 x *n* = 12 samples; NIV Fusarium vs. mock control).
