# Supplementary material for: Transcriptome Alterations of an in vitro-Selected, Moderately Resistant, Two-Row Malting Barley in Response to 3ADON, 15ADON, and NIV Chemotypes of Fusarium graminearum
Source: Front Plant Sci. 2021 Aug 11;12:701969. doi: 10.3389/fpls.2021.701969 (PMC8385242; doi:10.3389/fpls.2021.701969)
Supplement: Supplementary file 1 [file Data_Sheet_1.zip › Supplementary Figure S1.pdf]

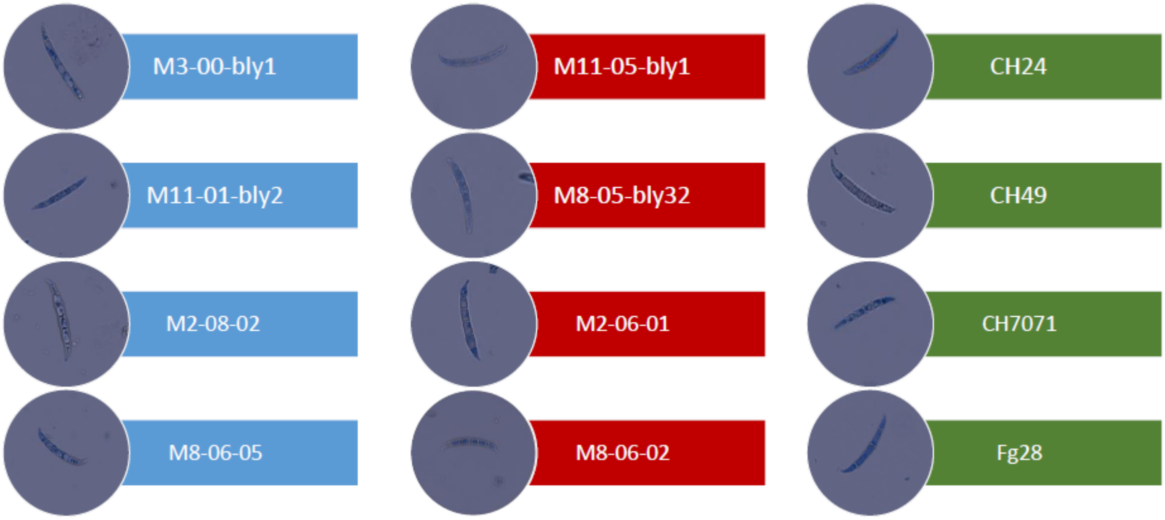

Isolate Code	CGC Code	NRRL code	Chemotype
WRS1915	M3-00-bly1	43162	15ADON
WRS1918	M11-01-bly2	43165	15ADON
M2-08-02	M2-08-02		15ADON
M8-06-05	M8-06-05		15ADON
WRS2065	M11-05-bly1	43217	3ADON
WRS2067	M8-05-bly32	43216	3ADON
M2-06-01	M2-06-01		3ADON
M8-06-02	M8-06-02		3ADON
Fg28			NIV
CH24			NIV
CH49			NIV
CH7071			NIV

CGC = Canadian Grain Commission; NRRL = United States Department of Agriculture, Agricultural Research Service (NRRL) collection.

Figure S1. *Fusarium graminearum* isolates and chemotype classification used in the study.
