# Supplementary material for: Transcriptome Alterations of an in vitro-Selected, Moderately Resistant, Two-Row Malting Barley in Response to 3ADON, 15ADON, and NIV Chemotypes of Fusarium graminearum
Source: Front Plant Sci. 2021 Aug 11;12:701969. doi: 10.3389/fpls.2021.701969 (PMC8385242; doi:10.3389/fpls.2021.701969)
Supplement: Supplementary file 1 [file Data_Sheet_1.zip › Supplementary Figure S3.pdf]

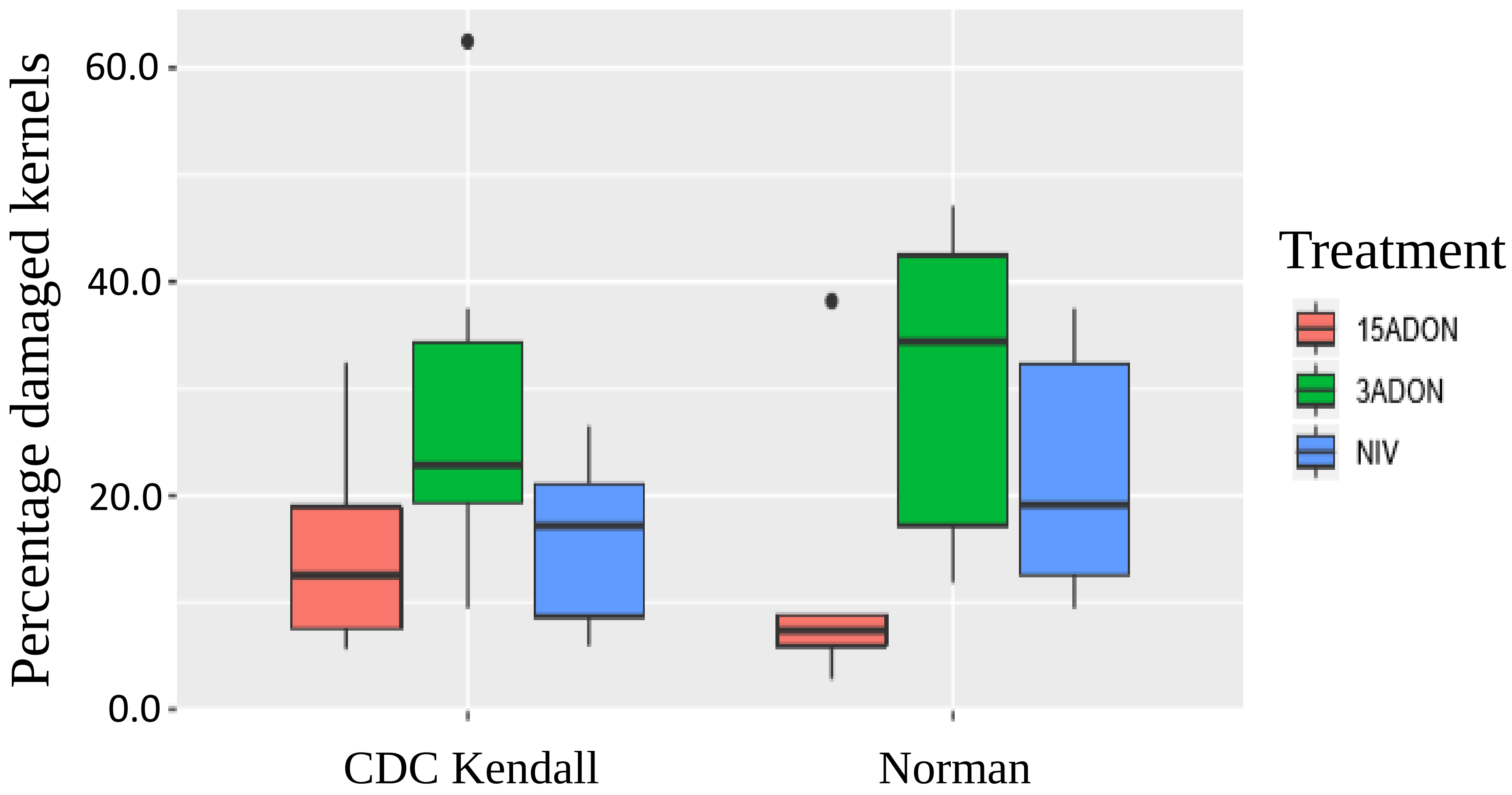

Figure S3. Percent Fusarium damaged kernels per spike over treatments: 15ADON (15-acetyldeoxynivalenol), 3ADON (3-acetyldeoxynivalenol), NIV (nivalenol) chemotypes by variety (n=6) in the growth cabinet study. No disease was observed under the mock treatment.
