# Supplementary material for: Transcriptome Alterations of an in vitro-Selected, Moderately Resistant, Two-Row Malting Barley in Response to 3ADON, 15ADON, and NIV Chemotypes of Fusarium graminearum
Source: Front Plant Sci. 2021 Aug 11;12:701969. doi: 10.3389/fpls.2021.701969 (PMC8385242; doi:10.3389/fpls.2021.701969)

Figure S5. Reads and GC content (%) per sample. R1 and R2 indicate forward and reverse strand respectively.

Number of reads per sample

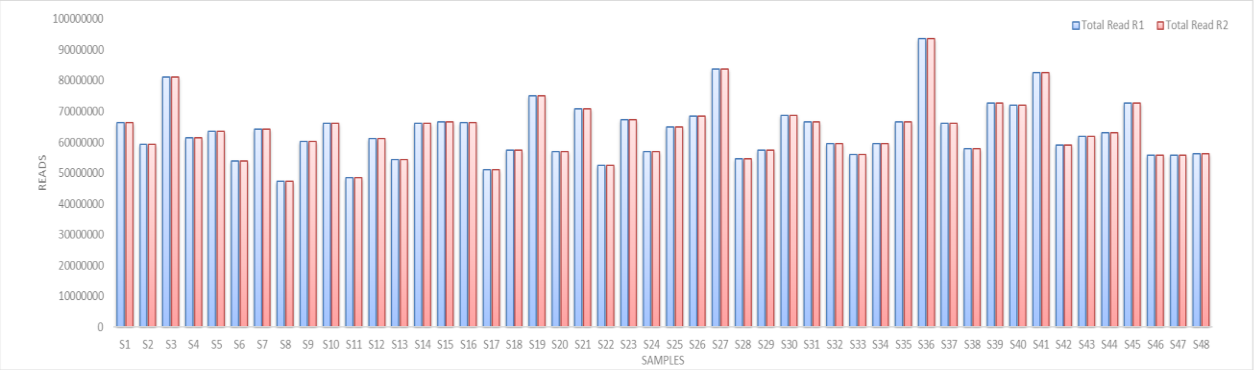

Sample ID	Total Read		Sample ID	Total Read		Sample ID	Total Read	
	R1	R2		R1	R2		R1	R2
S1	66246622	66246622	S17	51154954	51154954	S33	56109140	56109140
S2	59419800	59419800	S18	57396646	57396646	S34	59567586	59567586
S3	81109352	81109352	S19	75121255	75121255	S35	66677063	66677063
S4	61503028	61503028	S20	56986465	56986465	S36	93660519	93660519
S5	63546185	63546185	S21	70749856	70749856	S37	66019322	66019322
S6	53884500	53884500	S22	52514161	52514161	S38	57926516	57926516
S7	64127086	64127086	S23	67187185	67187185	S39	72710657	72710657
S8	47372468	47372468	S24	56900161	56900161	S40	71891982	71891982
S9	60177791	60177791	S25	65038296	65038296	S41	82634553	82634553
S10	66192463	66192463	S26	68519005	68519005	S42	59095659	59095659
S11	48478933	48478933	S27	83807875	83807875	S43	61836822	61836822
S12	61132880	61132880	S28	54561216	54561216	S44	63049737	63049737
S13	54380771	54380771	S29	57362755	57362755	S45	72641217	72641217
S14	66093056	66093056	S30	68754397	68754397	S46	55727719	55727719
S15	66560484	66560484	S31	66534392	66534392	S47	55807395	55807395
S16	66452389	66452389	S32	59562623	59562623	S48	56200520	56200520

GC Content per sample (%)

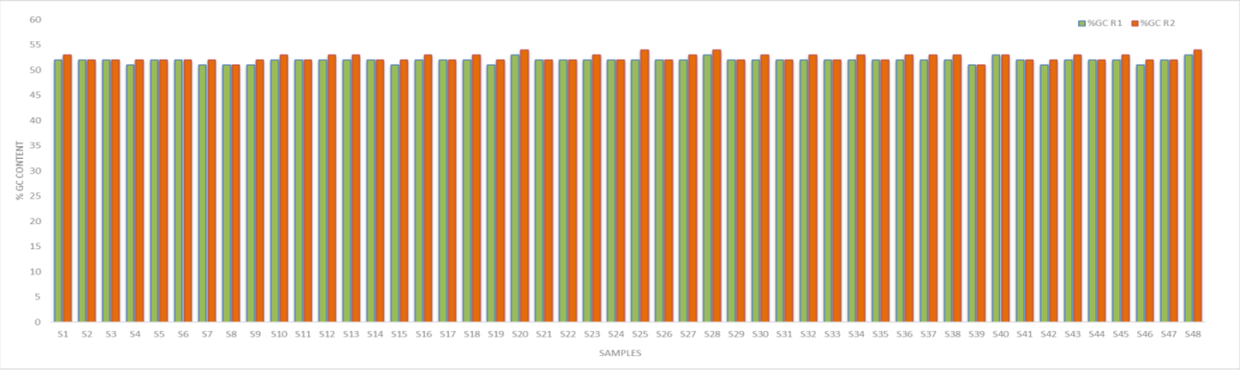

Supplement: Supplementary file 1 [file Data_Sheet_1.zip › Supplementary Figure S5.pdf]
