# Supplementary material for: Transcriptome Alterations of an in vitro-Selected, Moderately Resistant, Two-Row Malting Barley in Response to 3ADON, 15ADON, and NIV Chemotypes of Fusarium graminearum
Source: Front Plant Sci. 2021 Aug 11;12:701969. doi: 10.3389/fpls.2021.701969 (PMC8385242; doi:10.3389/fpls.2021.701969)
Supplement: Supplementary file 1 [file Data_Sheet_1.zip › Supplementary Figure S6.pdf]

Sample ID	R1	R2
**S1**	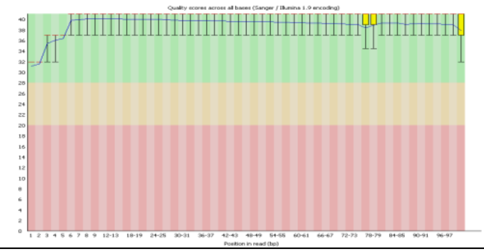	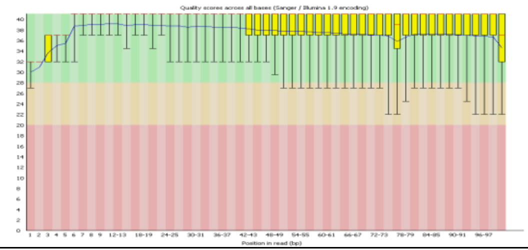
**S2**	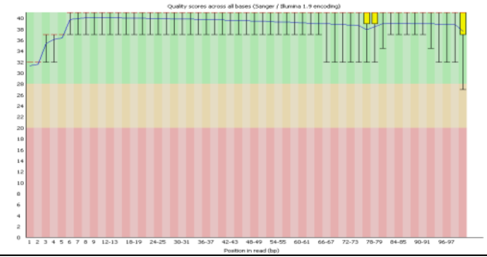	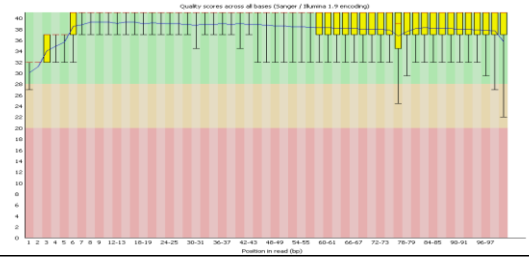
**S3**	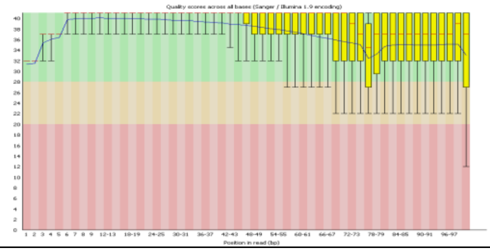	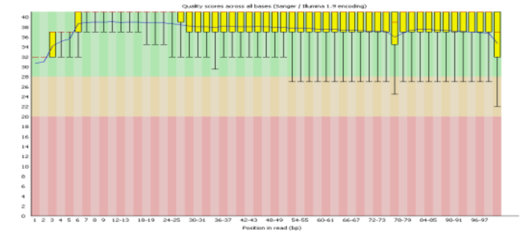
**S4**	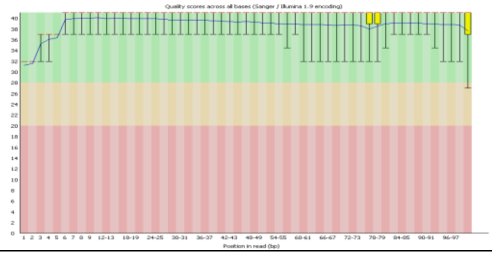	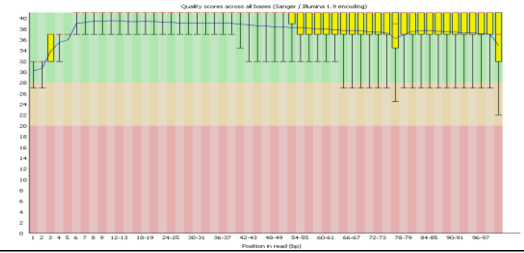
**S5**	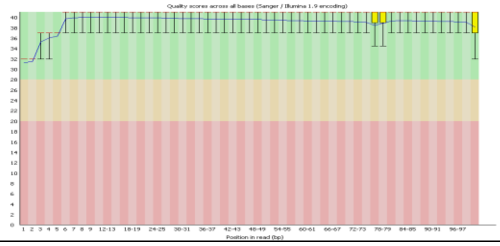	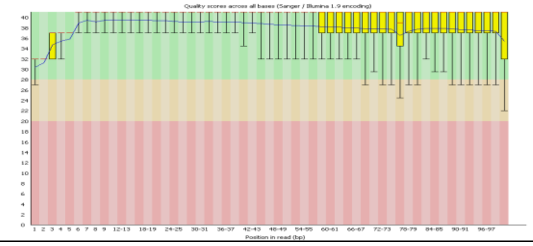
**S6**	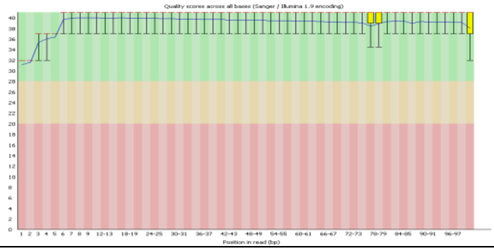	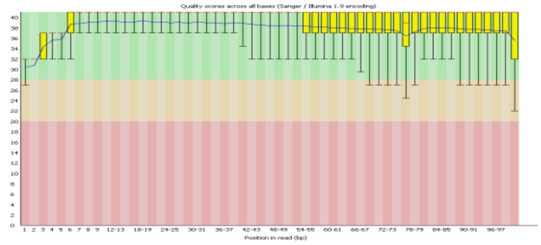

Figure S6: Continued

Sample ID	R1	R2
S7	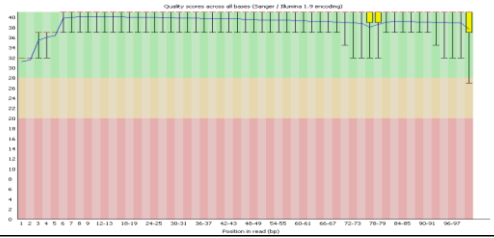	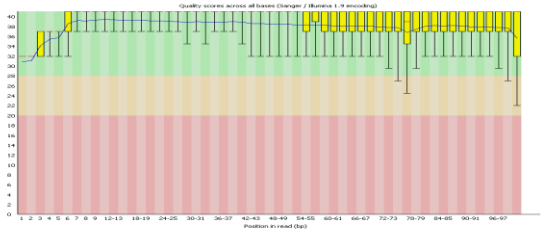
S8	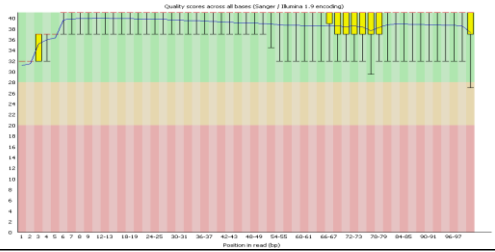	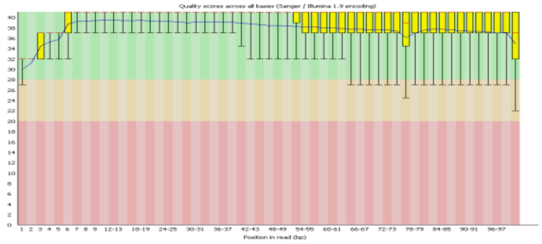
S9	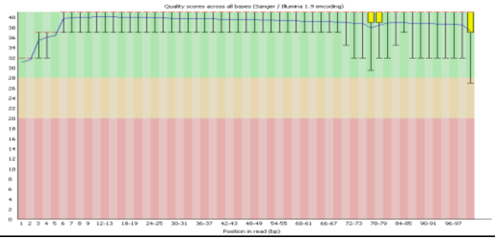	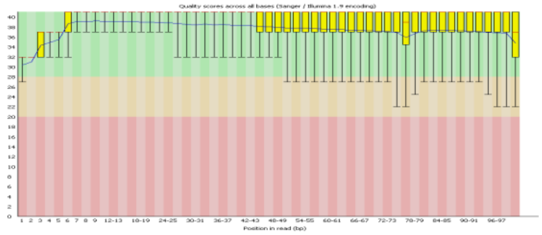
S10	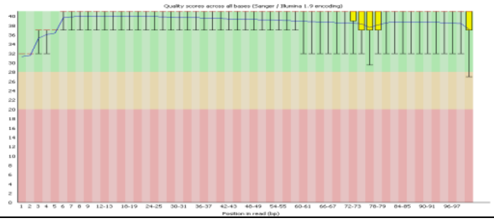	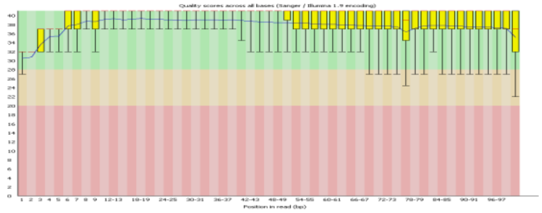
S11	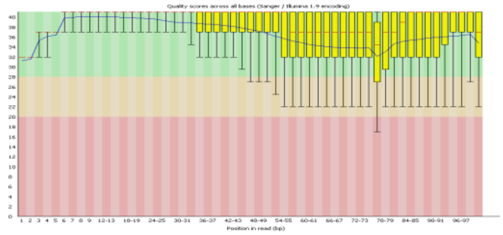	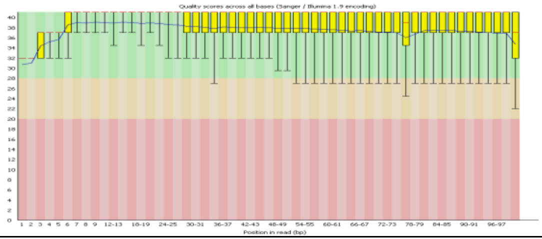
S12	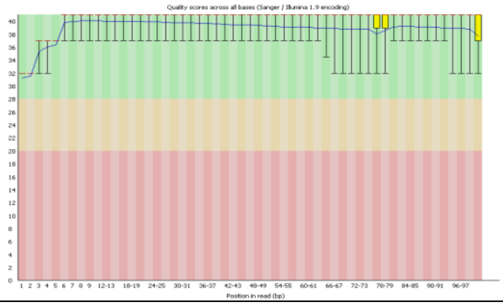	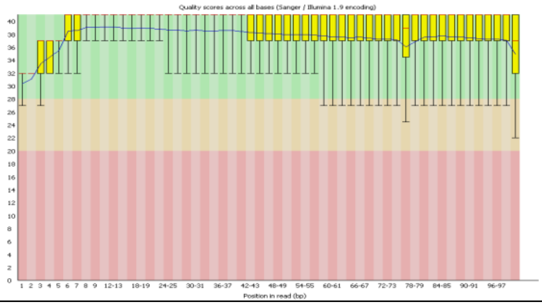

Figure S6: Continued

Sample ID	R1	R2
**S13**	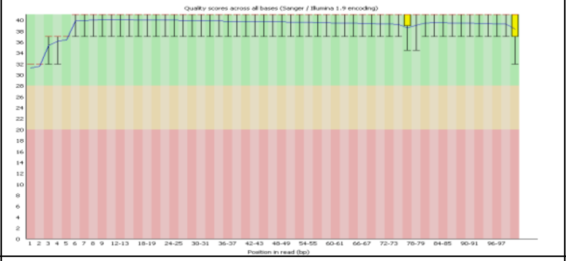	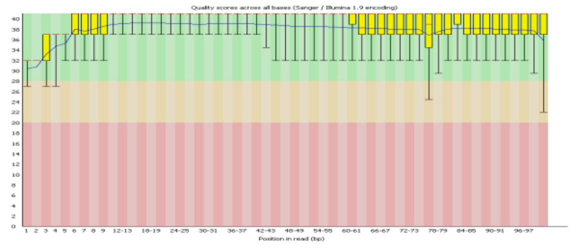
**S14**	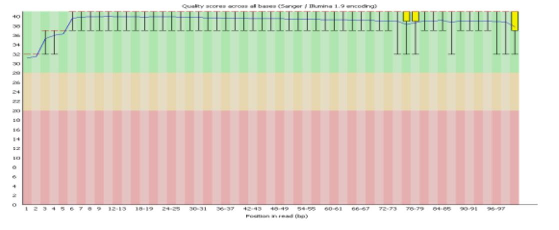	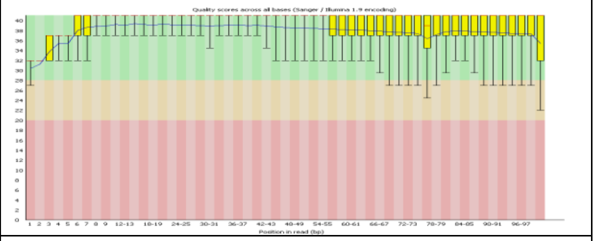
**S15**	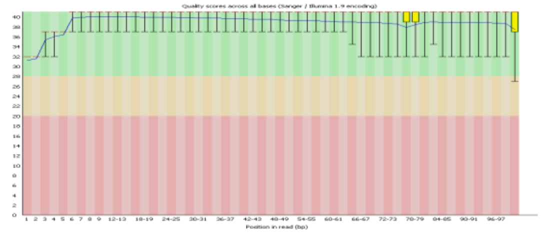	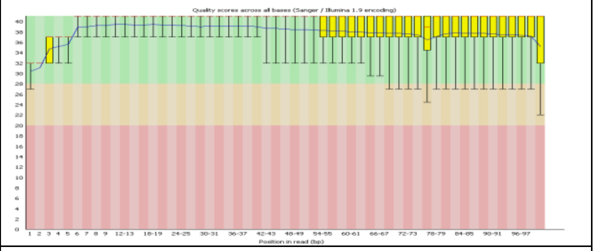
**S16**	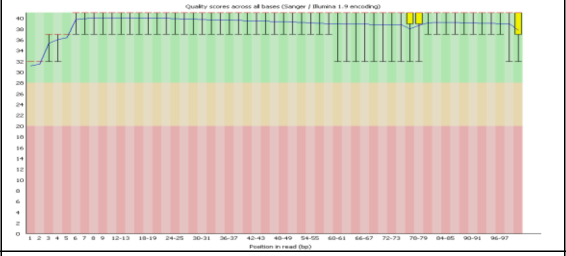	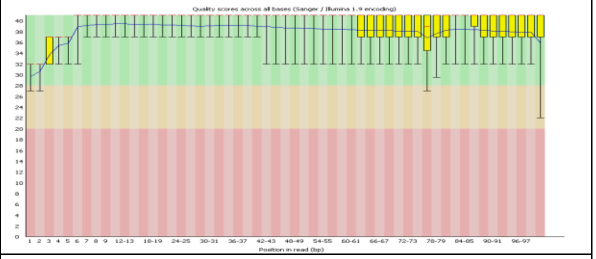
**S17**	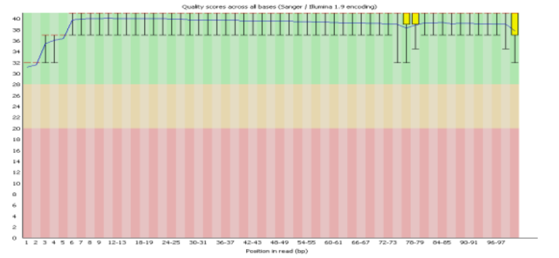	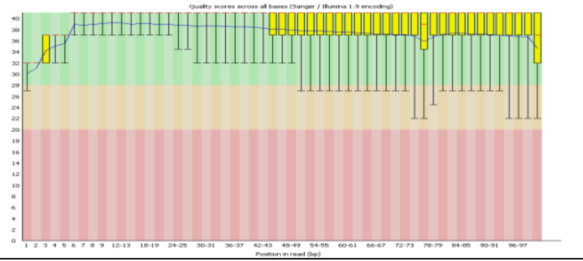
**S18**	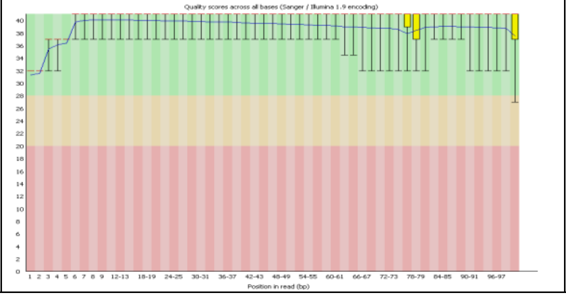	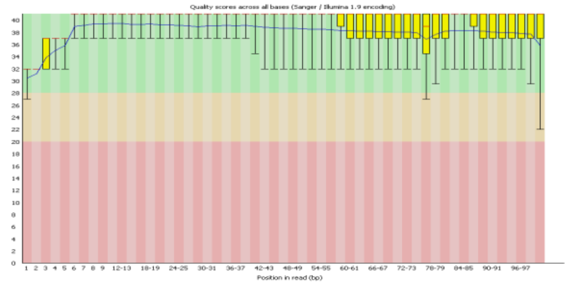

Figure S6: Continued

Sample ID	R1	R2
S19	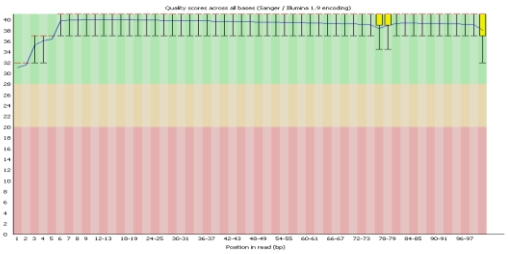	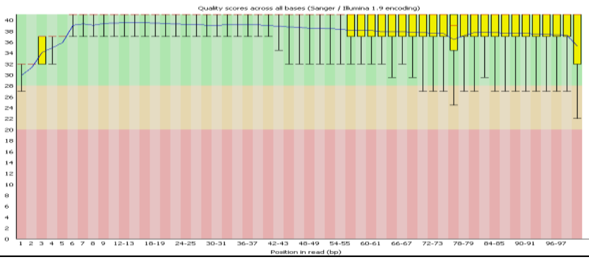
S20	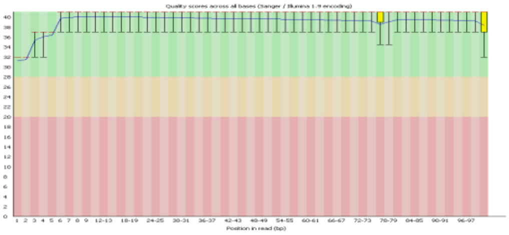	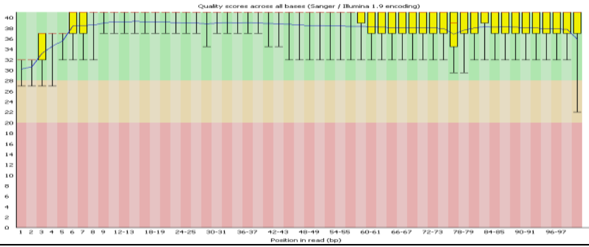
S21	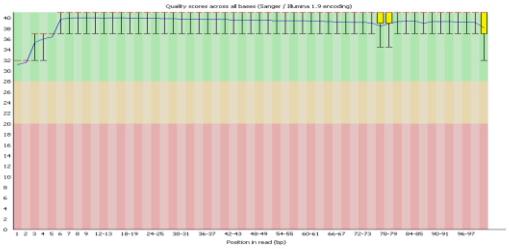	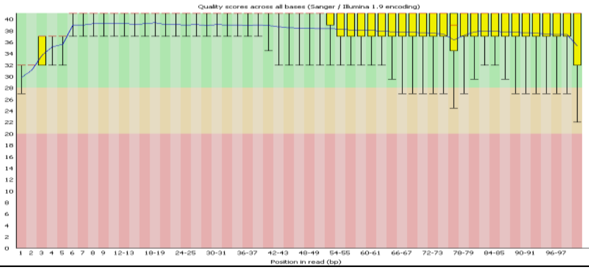
S22	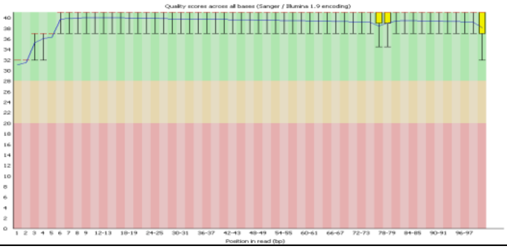	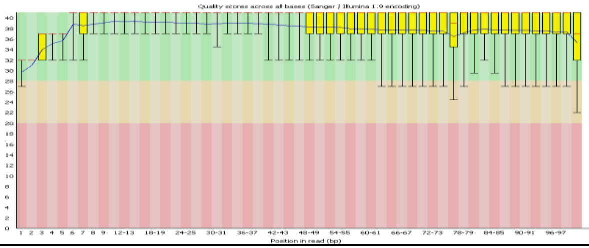
S23	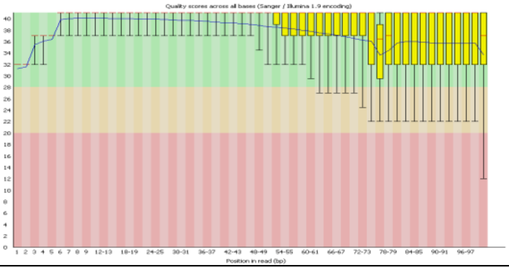	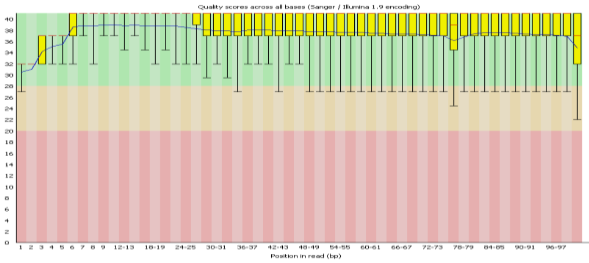
S24	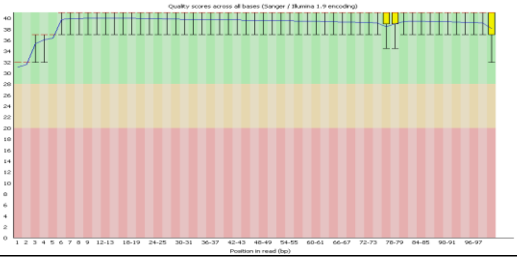	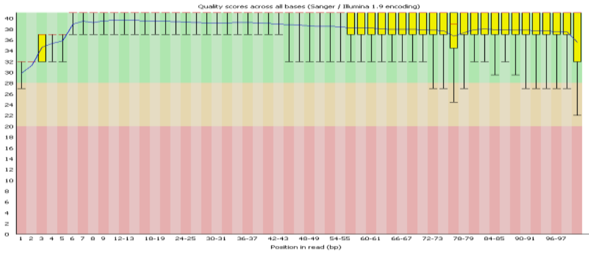

Figure S6: Continued

Sample ID	R1	R2
**S25**	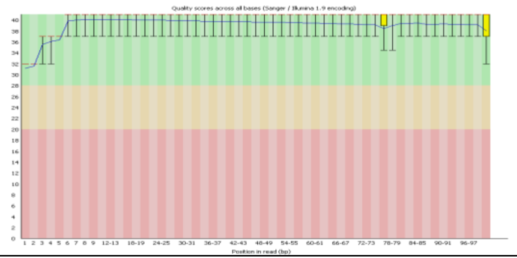	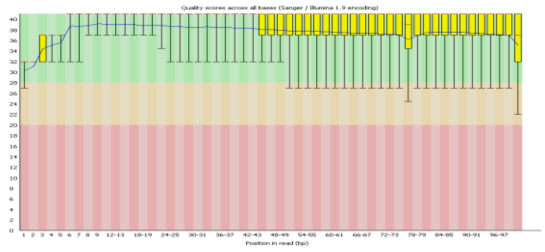
**S26**	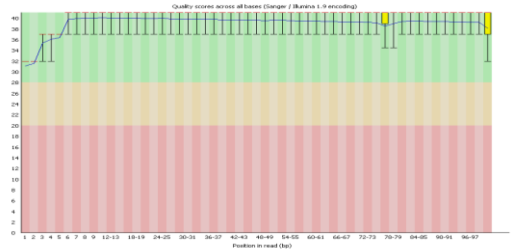	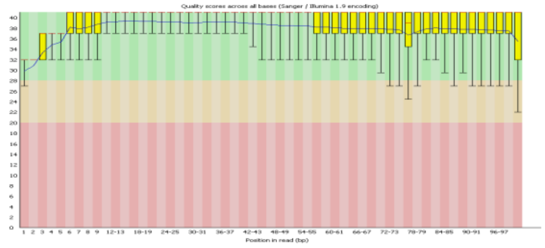
**S27**	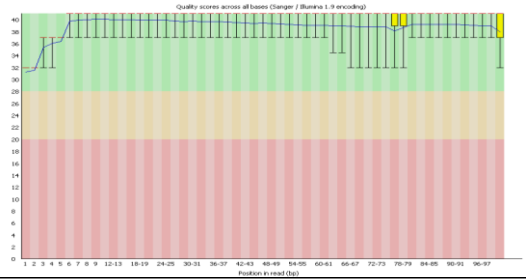	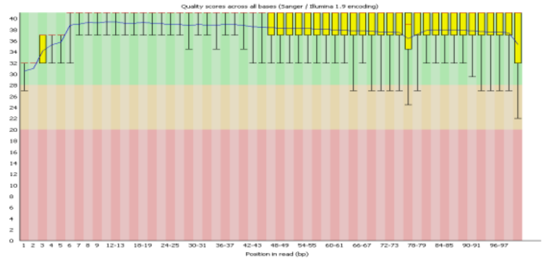
**S28**	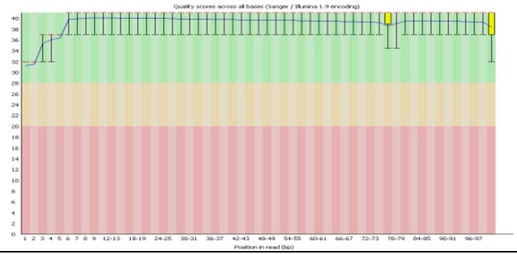	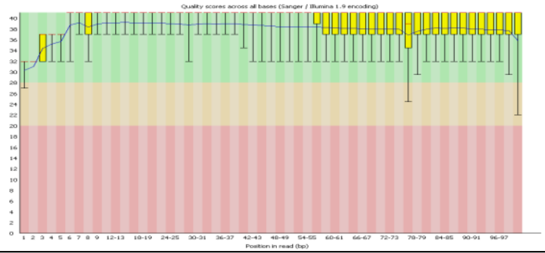
**S29**	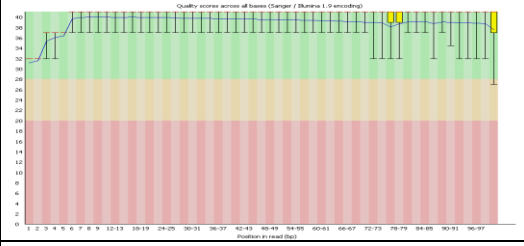	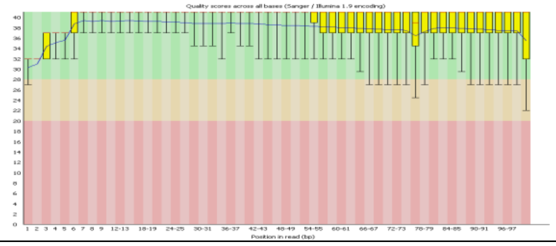
**S30**	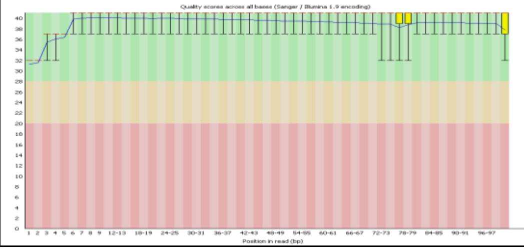	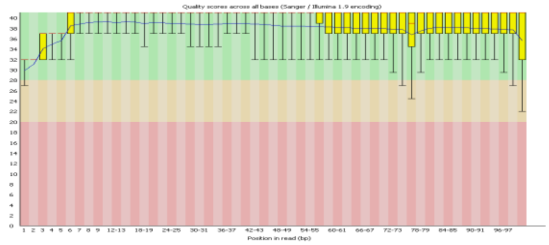

Figure S6: Continued

Sample ID	R1	R2
**S31**	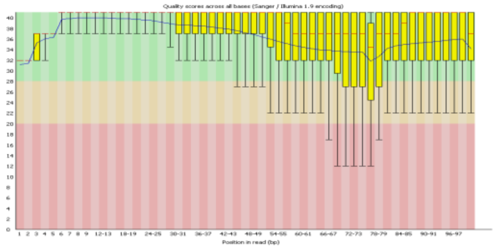	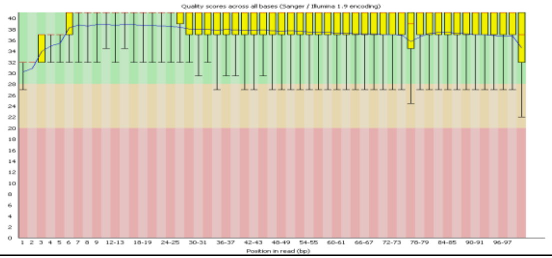
**S32**	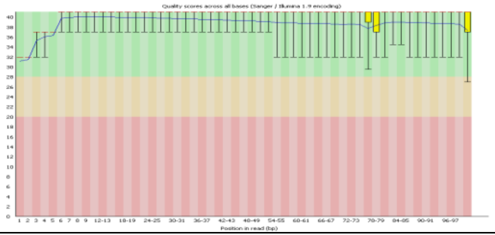	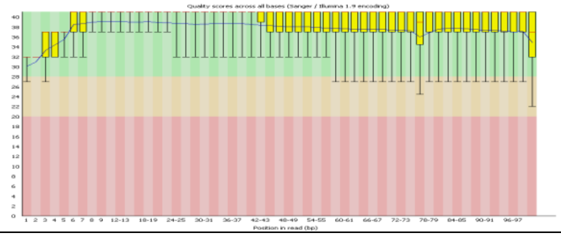
**S33**	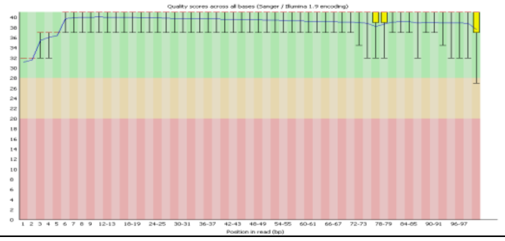	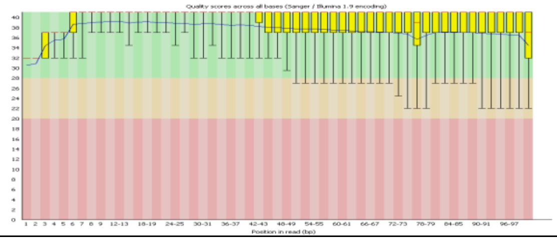
**S34**	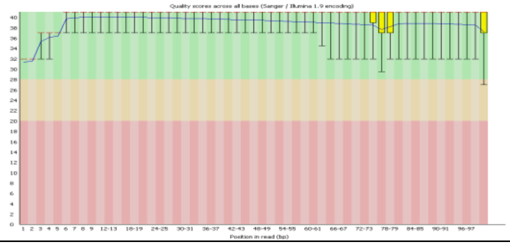	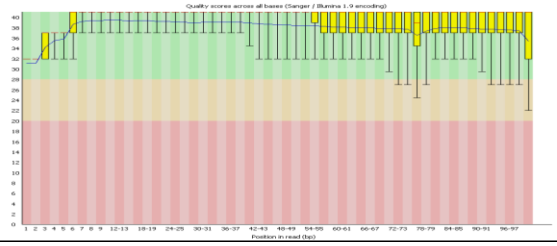
**S35**	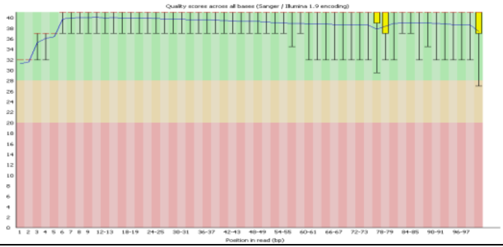	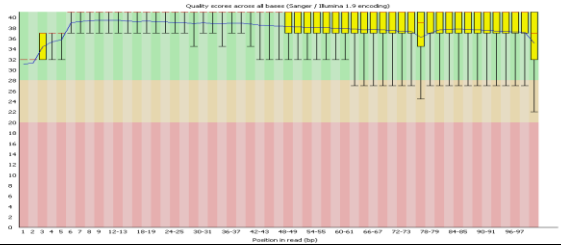
**S36**	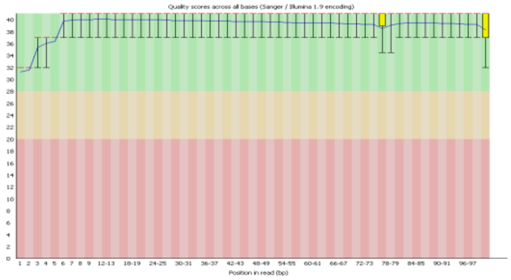	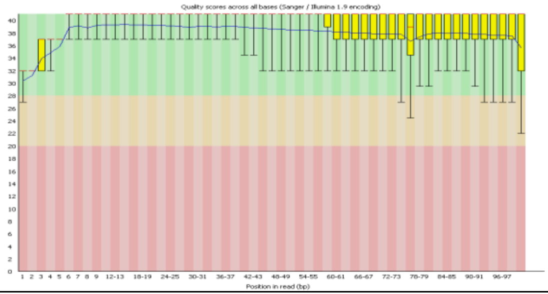

Figure S6: Continued

Sample ID	R1	R2
**S37**	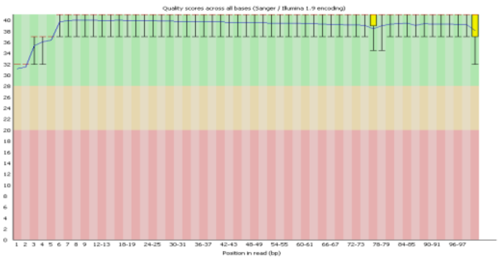	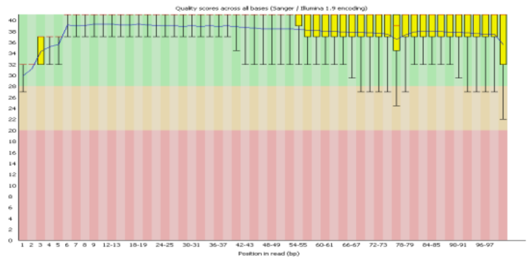
**S38**	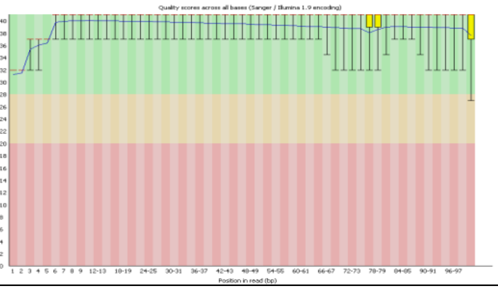	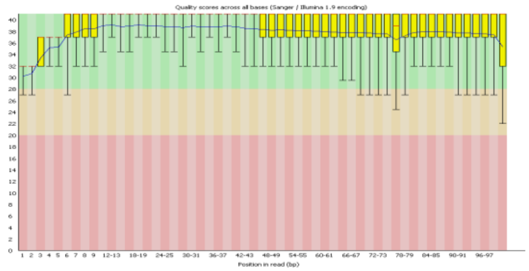
**S39**	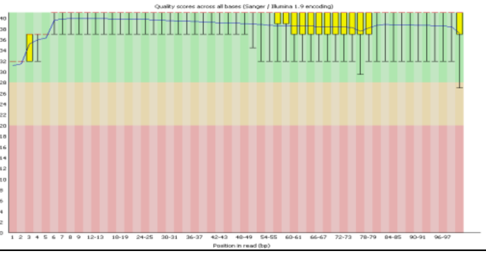	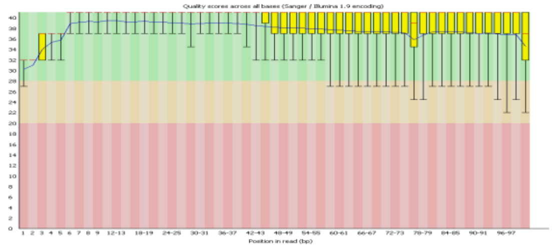
**S40**	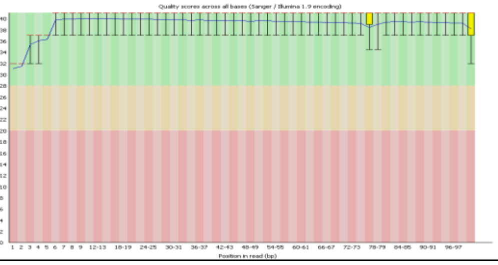	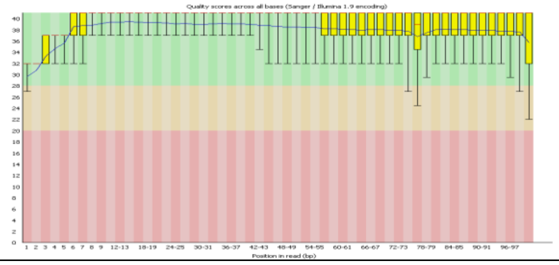
**S41**	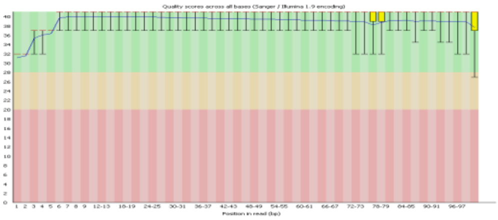	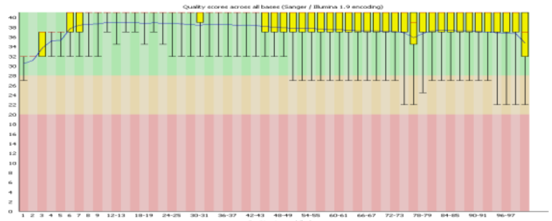
**S42**	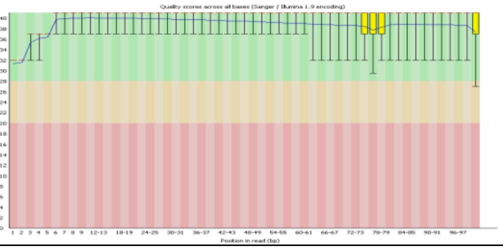	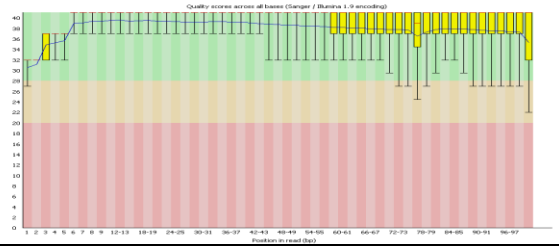

Figure S6: Continued

Sample ID	R1	R2
**S43**	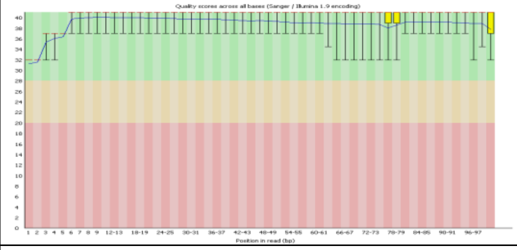	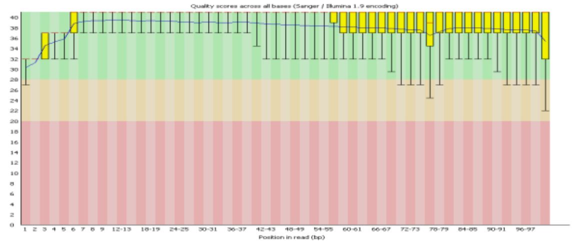
**S44**	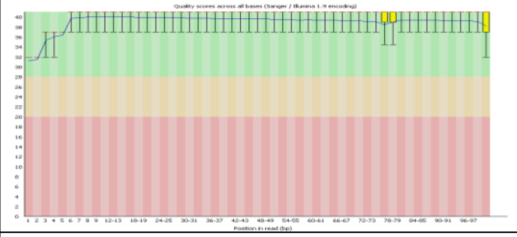	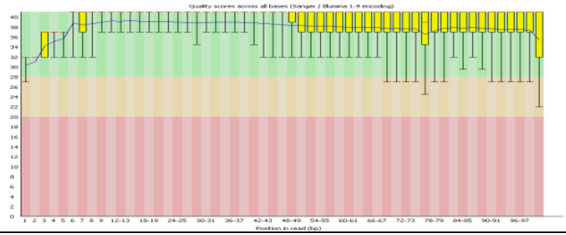
**S45**	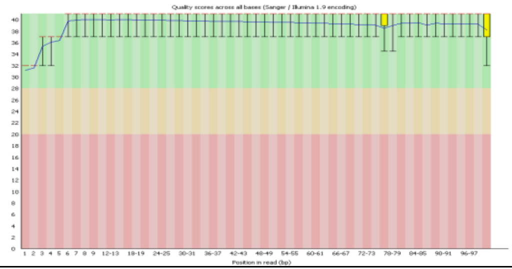	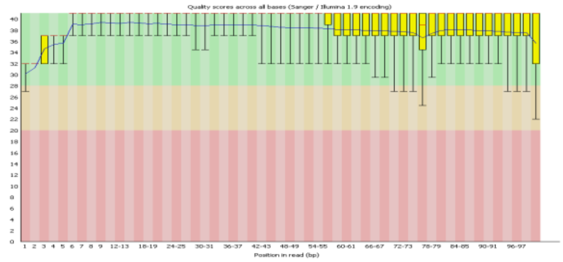
**S46**	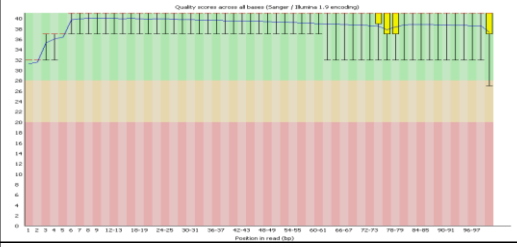	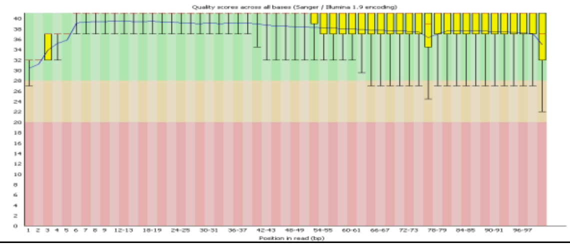
**S47**	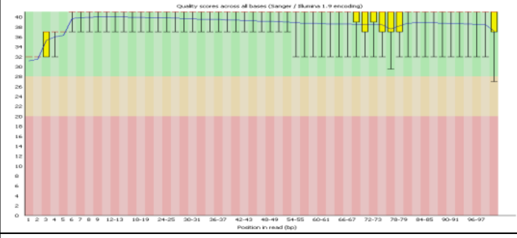	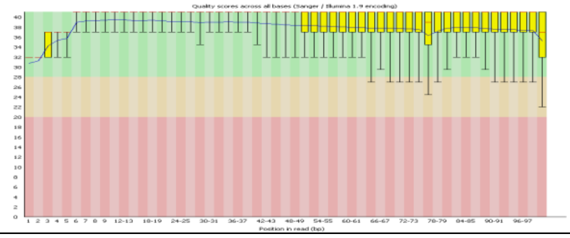
**S48**	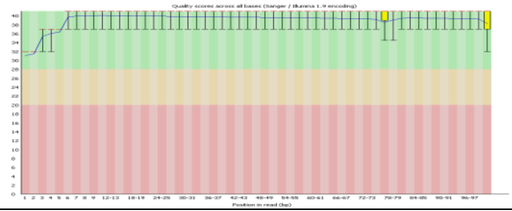	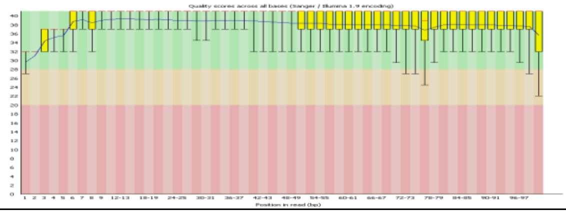

Figure S6. Read quality analysis from FastQC summary report for samples (1 to 48). Box-and-whisker plots of quality scores by position of read. R1 & R2 indicates forward and reverse strand respectively.
