# Supplementary material for: Transcriptome Alterations of an in vitro-Selected, Moderately Resistant, Two-Row Malting Barley in Response to 3ADON, 15ADON, and NIV Chemotypes of Fusarium graminearum
Source: Front Plant Sci. 2021 Aug 11;12:701969. doi: 10.3389/fpls.2021.701969 (PMC8385242; doi:10.3389/fpls.2021.701969)
Supplement: Supplementary file 1 [file Data_Sheet_1.zip › Supplementary Table S1.pdf]

**Table S1.** Percent (%) alignment by sample to barley reference genome (IBSC_v2) using HISAT2.

| Sample_ID | Percent (%) | TIME | VARIETY | TREAT | REP |
|---|---|---|---|---|---|
| 1 | 92.7 | 72 | N | mock | 1 |
| 2 | 90.8 | 72 | K | mock | 1 |
| 5 | 91.8 | 72 | N | mock | 2 |
| 6 | 87.3 | 72 | K | mock | 2 |
| 11 | 90.9 | 72 | N | mock | 3 |
| 12 | 93.3 | 72 | K | mock | 3 |
| 3 | 87.5 | 96 | K | mock | 1 |
| 4 | 90.5 | 96 | N | mock | 1 |
| 7 | 93.9 | 96 | K | mock | 2 |
| 8 | 90.2 | 96 | N | mock | 2 |
| 9 | 91.5 | 96 | K | mock | 3 |
| 10 | 89.3 | 96 | N | mock | 3 |
| 16 | 92.5 | 72 | K | 15ADON | 1 |
| 17 | 90.6 | 72 | K | 15ADON | 2 |
| 23 | 92.9 | 72 | K | 15ADON | 3 |
| 15 | 92.5 | 72 | N | 15ADON | 1 |
| 18 | 91.3 | 72 | N | 15ADON | 2 |
| 24 | 81.1 | 72 | N | 15ADON | 3 |
| 14 | 82.4 | 96 | K | 15ADON | 1 |
| 19 | 87.5 | 96 | K | 15ADON | 2 |
| 22 | 90.7 | 96 | K | 15ADON | 3 |
| 13 | 89.1 | 96 | N | 15ADON | 1 |
| 20 | 75.9 | 96 | N | 15ADON | 2 |
| 21 | 92.2 | 96 | N | 15ADON | 3 |
| 28 | 90.5 | 72 | K | 3ADON | 1 |
| 30 | 92.7 | 72 | K | 3ADON | 2 |
| 35 | 91 | 72 | K | 3ADON | 3 |
| 27 | 92.6 | 72 | N | 3ADON | 1 |
| 29 | 92 | 72 | N | 3ADON | 2 |
| 33 | 89.6 | 72 | N | 3ADON | 3 |
| 25 | 92.5 | 96 | K | 3ADON | 1 |
| 32 | 85.2 | 96 | K | 3ADON | 2 |
| 34 | 86.9 | 96 | K | 3ADON | 3 |
| 26 | 88.3 | 96 | N | 3ADON | 1 |
| 31 | 91.4 | 96 | N | 3ADON | 2 |
| 36 | 90.9 | 96 | N | 3ADON | 3 |
| 38 | 90.9 | 72 | K | NIV | 1 |
| 42 | 88.7 | 72 | K | NIV | 2 |
| 45 | 91.6 | 72 | K | NIV | 3 |
| 37 | 92.7 | 72 | N | NIV | 1 |
| 41 | 90 | 72 | N | NIV | 2 |
| 46 | 89.3 | 72 | N | NIV | 3 |
| 40 | 83.7 | 96 | K | NIV | 1 |
| 43 | 91.6 | 96 | K | NIV | 2 |
| 47 | 89.8 | 96 | K | NIV | 3 |
| 39 | 87.2 | 96 | N | NIV | 1 |
| 44 | 91.2 | 96 | N | NIV | 2 |
| 48 | 85.6 | 96 | N | NIV | 3 |

Time: 72 & 96 hours post infection; Variety: N = Norman & K = CDC Kendall; Treat: mock = control; 15ADON = 15-acetyldeoxynivalenol, 3ADON = 3-acetyldeoxynivalenol, NIV = nivalenol.
