# Supplementary material for: Transcriptome Alterations of an in vitro-Selected, Moderately Resistant, Two-Row Malting Barley in Response to 3ADON, 15ADON, and NIV Chemotypes of Fusarium graminearum
Source: Front Plant Sci. 2021 Aug 11;12:701969. doi: 10.3389/fpls.2021.701969 (PMC8385242; doi:10.3389/fpls.2021.701969)
Supplement: Supplementary file 1 [file Data_Sheet_1.zip › Supplementary Table S2.pdf]

Table S2. Analysis of variance (ANOVA) for proportion of *Fusarium* damaged kernels and Tukey-Kramer groups for chemotype treatment.

ANOVA – Type III Tests of Fixed Effects				
**Effect**	**Num DF**	**Den DF**	**F value**	**Pr>F**
**Variety**	1	30	0.06	0.803
**Treatment**	2	30	4.82	0.015
**Variety x Treatment**	2	30	0.45	0.643

Tukey-Kramer Grouping for Chemotype Treatment (α=0.05)

Treatment	FDK
**3ADON**	0.30 a
**NIV**	0.19 ab
**15ADON**	0.13 b

FDK = Proportion of *Fusarium* damaged kernels ; LS-means with the same letter are not significantly different.
