# Supplementary material for: Transcriptome Alterations of an in vitro-Selected, Moderately Resistant, Two-Row Malting Barley in Response to 3ADON, 15ADON, and NIV Chemotypes of Fusarium graminearum
Source: Front Plant Sci. 2021 Aug 11;12:701969. doi: 10.3389/fpls.2021.701969 (PMC8385242; doi:10.3389/fpls.2021.701969)
Supplement: Supplementary file 1 [file Data_Sheet_1.zip › Supplementary Table S3.pdf]

Table S3. Analysis of variance (ANOVA) for content of primary mycotoxin (DON or NIV)

ANALYSIS OF VARIANCE (ANOVA) Type III Tests of Fixed Effects				
**Effect**	**Num DF**	**Den DF**	**F Value**	**Pr > F**
**Variety**	1	6	0.02	0.90
**Treatment**	2	6	12.63	0.01
**Variety*Treatment**	2	6	0.08	0.92
