# Supplementary material for: Transcriptome Alterations of an in vitro-Selected, Moderately Resistant, Two-Row Malting Barley in Response to 3ADON, 15ADON, and NIV Chemotypes of Fusarium graminearum
Source: Front Plant Sci. 2021 Aug 11;12:701969. doi: 10.3389/fpls.2021.701969 (PMC8385242; doi:10.3389/fpls.2021.701969)
Supplement: Supplementary file 1 [file Data_Sheet_1.zip › Supplementary Table S4.pdf]

Table S4. Percent variant markers by chromosome

Chr[1]	Total SNP markers	Variant markers	Variance (%)
0	6251	171	2.7
1H	3468	251	5.9
2H	6168	2	0.0
3H	5803	1	0.0
4H	4480	76	1.7
5H	6930	67	1.0
6H	4678	0	0.0
7H	5515	527	9.6
**Total**	**39363**	**1095**	**1.8**

[1]Chromosome assignment as per Bayer et al. (2017) ; 0 = Position unassigned.
